# Dysfunctional cerebellar Purkinje cells contribute to autism-like behaviour in *Shank2*-deficient mice

**DOI:** 10.1038/ncomms12627

**Published:** 2016-09-01

**Authors:** Saša Peter, Michiel M. ten Brinke, Jeffrey Stedehouder, Claudia M. Reinelt, Bin Wu, Haibo Zhou, Kuikui Zhou, Henk-Jan Boele, Steven A. Kushner, Min Goo Lee, Michael J. Schmeisser, Tobias M. Boeckers, Martijn Schonewille, Freek E. Hoebeek, Chris I. De Zeeuw

**Affiliations:** 1 Netherlands Institute for Neuroscience, Amsterdam 1105 CA, Netherlands; 2 Department of Neuroscience, Erasmus MC, Rotterdam 3000 DR, Netherlands; 3 Department of Psychiatry, Erasmus MC, Rotterdam 3000 DR, Netherlands; 4 Institute for Anatomy and Cell Biology, Ulm University, Ulm 89081, Germany; 5 Yonsei University College of Medicine, Seoul 120–752, Korea; 6 Department of Neurology, Ulm University, Ulm 89081, Germany

## Abstract

Loss-of-function mutations in the gene encoding the postsynaptic scaffolding protein *SHANK2* are a highly penetrant cause of autism spectrum disorders (ASD) involving cerebellum-related motor problems. Recent studies have implicated cerebellar pathology in the aetiology of ASD. Here we evaluate the possibility that cerebellar Purkinje cells (PCs) represent a critical locus of ASD-like pathophysiology in mice lacking *Shank2*. Absence of *Shank2* impairs both PC intrinsic plasticity and induction of long-term potentiation at the parallel fibre to PC synapse. Moreover, inhibitory input onto PCs is significantly enhanced, most prominently in the posterior lobe where simple spike (SS) regularity is most affected. Using PC-specific *Shank2* knockouts, we replicate alterations of SS regularity *in vivo* and establish cerebellar dependence of ASD-like behavioural phenotypes in motor learning and social interaction. These data highlight the importance of *Shank2* for PC function, and support a model by which cerebellar pathology is prominent in certain forms of ASD.

## Abstract (web summary)

Mutations in *SHANK2* are associated with autism spectrum disorders (ASD). Here, Peter *et al.* show that selective loss of *Shank2* in Purkinje cells of the mouse cerebellum leads to deficits in plasticity, motor behaviour, and a social behaviour phenotype similar to that seen in ASD.

## Graphical abstract

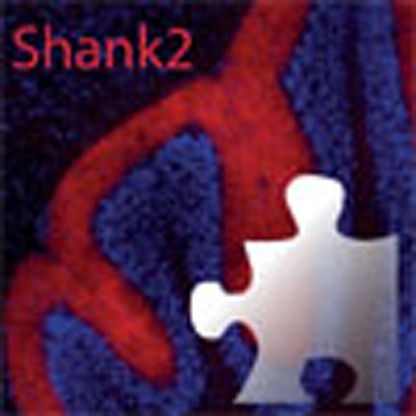

Autism spectrum disorders (ASD) are neurodevelopmental disease entities primarily defined by deficits in social interaction and repetitive behaviour^1^. In addition, individuals with autism often suffer from motor skill deficiencies^2^, many of which manifest early in the disease^3^. The aetiology of ASD is complex with reported pathophysiological alterations encompassing multiple brain regions, including the cerebellum^1^. Cerebellum-related motor symptoms of ASD patients have been observed through impairments in eye-blink conditioning^4^,^5^, eye movement abnormalities^6^,^7^, general motor learning deficits^8^,^9^, as well as balance and postural difficulties^10^,^11^. Patients with cerebellar lesions emerging later in development are often diagnosed with cerebellar cognitive affective syndrome, a condition characterized by deficits in language, executive function and emotion regulation which overlaps considerably with symptoms in ASD^12^. Anatomical evidence for cerebellar involvement in ASD includes a decrease in the number of Purkinje cells (PCs) by post-mortem brain histopathological examination^13^,^14^ and functional connectivity between the cerebellum, and frontoparietal and sensorimotor regions in resting-state fMRI studies of ASD^15^. Moreover, the cerebellum is among the most prominent brain regions demonstrating high co-expression of ASD-associated genes^16^.

Emerging data indicate that neurodevelopmental disorders including ASD result from dysfunctional synaptic networks^17^,^18^. The postsynaptic density (PSD) in particular represents a critically important proteomic hub for a considerable proportion of mutations causing neurodevelopmental diseases, including ASD^19^. A prominent example is the Shank family of postsynaptic scaffolding proteins, which has gained wide attention because of their strong link to ASD^20^,^21^,^22^,^23^,^24^. To date, two studies have independently reported generating *Shank2* knockout (KO) mice with ASD-like behaviour and abnormal hippocampal processing^25^,^26^. However, beyond the forebrain, *Shank2* is also highly expressed in cerebellar PCs^27^,^28^. Moreover, patients with *SHANK2*-related ASD exhibit motor impairments consistent with cerebellar dysfunction^29^. However, the causal influence of cerebellar dysfunction on *Shank2*-related models of ASD has never been established.

We therefore used both global germ-line *Shank2* KO (*Shank2*^−/−^) and PC-specific *Shank2* KO (*L7-Shank2*^−/−^) mouse models to investigate the causal influence of *Shank2* on cerebellar function and ASD-related behaviours. Notably, *Shank2*^−/−^ mice have impairments in plasticity at the parallel fibre (PF) to PC synapse, increased inhibitory input onto PCs and significant irregularities in PC simple spike (SS) activity. Moreover, *L7-Shank2*^−/−^ mice show deficits in social interaction and exhibit task-specific repetitive behaviour. Together, these results provide novel insight into the pathophysiological mechanisms by which *SHANK2* mutations cause impairments in cerebellar function that may contribute to ASD.

## Results

### Decreased AMPAR in *Shank2* ^−*/*−^ cerebellar synaptosomes

**Figure 1:**
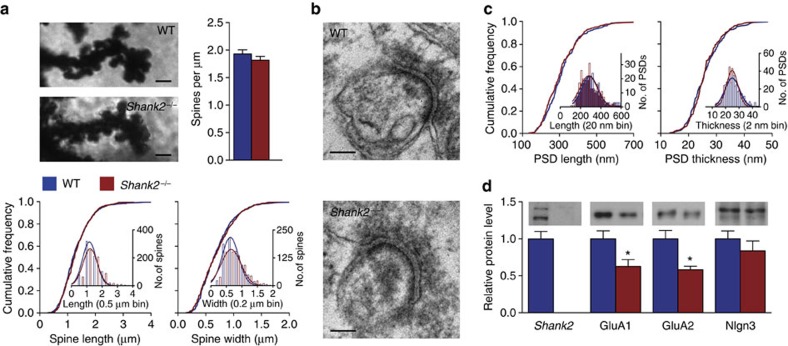
Reduction of AMPA receptor subunits in *Shank2*^−/−^ cerebellar synaptosomes in the absence of changes in spine and PSD morphology in the DML. (**a**) Representative images (Golgi-Cox staining) of distal Purkinje cell dendrites in the distal molecular layer (DML), quantification of spine density (WT, *n*=97/4, dendrites per mice; *Shank2*^−/−^, *n*=89/4, *P*=0.2, MWU-test) and cumulative frequency plots of spine length (*P*=0.4, MWU-test) and thickness (*P*=1, MWU-test) in WT (*n*=748/4 spines per mice) and *Shank2*^−/−^ mice (*n*=639/4) as indicated. Scale bar, 1 μm. (**b**,**c**) Representative images (electron microscopy) of spine synapses in the DML and cumulative frequency plots of PSD length (WT, *n*=226/4, PSDs per mice; *Shank2*^−/−^, *n*=243/4, *P*=0.3) and thickness (WT, *n*=223/4; *Shank2*^−/−^, *n*=233/4, *P*=0.9) as indicated. Scale bar, 100 nm. (**d**) Biochemical analysis of *Shank2* (WT, *n*=12 synaptosomes; *Shank2*^−/−^, *n*=6), GluA1 (WT, *n*=12 synaptosomes; *Shank2*^−/−^, *n*=6, *P*=0.041) GluA2 (WT, *n*=11; *Shank2*^−/−^, *n*=5, *P*=0.014) and *Nlgn3* (WT, *n*=12; *Shank2*^−/−^, *n*=6, *P*=0.4) in cerebellar synaptosomes from WT and *Shank2*^−/−^ mice as indicated. Data in bar graphs are presented as mean±s.e.m.; single asterisks indicates *P*<0.05. Two-sided *t*-tests were used, unless stated otherwise.

A divergent role of the SHANK2 scaffolding protein has been hypothesized for PSD function and cellular morphology^30^. To evaluate the morphology of *Shank2*-deficient postsynaptic specializations along PC dendrites, we quantified the structural characteristics of dendritic spines and PSDs in the distal molecular layer of global *Shank2*^−/−^ mice using Golgi-Cox staining of PC dendrites and electron microscopy (Fig. 1). Neither spine density (WT: 1.93±0.74 spines per μm dendrite; *Shank2*^−/−^: 1.82±0.67 spines per μm dendrite; *P*=0.2, Mann–Whitney *U*-test, MWU-test, see Supplementary Table 1 for additional statistics), nor the length (WT: 1.34±0.77 μm; *Shank2*^−/−^: 1.32±0.50 μm; *P*=0.4, MWU-test) or width of individual spines (WT: 0.72±0.45 μm; *Shank2*^−/−^: 0.71±0.32 μm; *P*=0.9, MWU-test) was significantly affected (Fig. 1a). In addition, the length (WT: 313.3±97.1 nm; *Shank2*^−/−^: 305.3±96.2 nm; *P*=0.3) and thickness of PSDs (WT: 26.2±6.0 nm; *Shank2*^−/−^: 26.0±5.3 nm; *P*=0.9) were similar between genotypes (Fig. 1b,c). In contrast, biochemical analysis of cerebellar synaptosomes indicated that global *Shank2*^−/−^ mice have lowered expression of AMPA receptor subunits GluA1 (WT: 1.00±0.37; *Shank2*^−/−^: 0.63±0.23; *P*=0.041) (Fig. 1d, Supplementary Fig. 1) and GluA2 (WT: 1.00±0.32; *Shank2*^−/−^: 0.58±0.11; *P*=0.014). In addition, we looked into the ASD pathology-related cell adhesion molecule neuroligin 3 (Nlgn3), which has been shown to interact with Shank proteins^19^, but found no significant difference in its expression (WT: 1.00±0.37; *Shank2*^−/−^ 0.84±0.33; *P*=0.4). Together, these findings indicate that *Shank2* is not crucial for the morphological differentiation of PC dendritic spines and PSDs, but instead may play an important role in the maintenance of cerebellar GluA1 and GluA2 levels.

### Normal baseline excitability in *Shank2* ^−/−^ PCs

**Figure 2:**
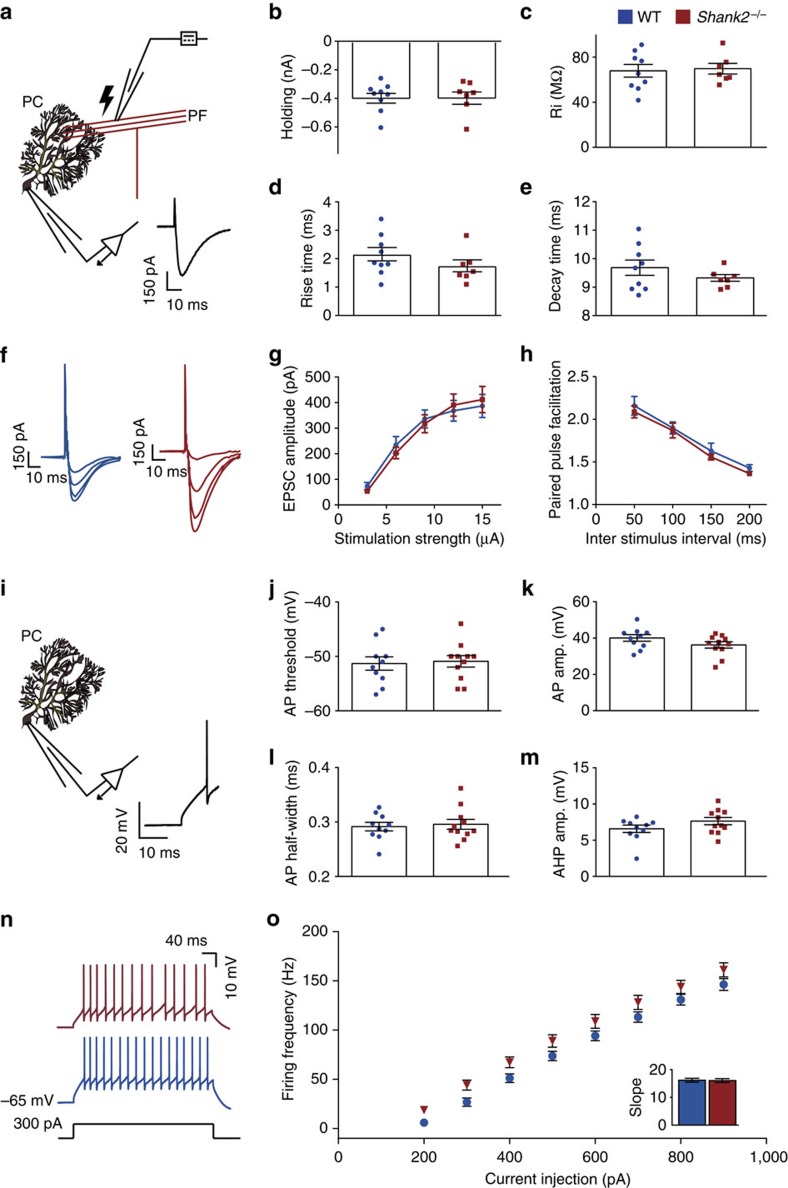
No changes in excitatory synaptic and intrinsic properties in *Shank2*^−/−^ Purkinje cells *ex vivo*. (**a**) Recording configuration for voltage-clamp recordings of PF–PC synaptic transmission. Inset: an example PF-EPSC. (**b**–**e**) With comparable holding current (at −65 mV) (*P*=1) and input resistance (Ri) (*P*=0.8), PC EPSC rise time (*P*=0.2) and EPSC decay time (*P*=0.3) are not different between WT (*n*=9/6, cells per animals) and *Shank2*^−/−^ (*n*=7/6). (**f**) Example EPSCs in response to 3, 6, 9, 12 and 15 μA stimulation. (**g**,**h**) Varying stimulation strength (*P*=0.9, repeated-measures ANOVA) and inter-stimulus interval (*P*=0.2, repeated-measures ANOVA) evoked comparable EPSC amplitude or facilitation (WT, *n*=11/3; *Shank2*^−/−^ 15/3). (**i**) Recording configuration for whole-cell recording. Inset: an example action potential. (**j**–**m**) Action potential threshold (*P*=0.8), amplitude (*P*=0.1), half-width (*P*=0.7) and after-hyperpolarization (*P*=0.2) were not different (WT, *n*=10/6; *Shank2*^−/−^, *n*=11/6). (**n**) Example traces of intrinsic Purkinje cell excitability as apparent from action potential firing evoked by 300 pA current injections. (**o**) No difference in evoked firing frequency relative to various levels of current injections (WT, *n*=10/5; *Shank2*^−/−^, *n*=11/5, *P*=0.1, repeated-measures ANOVA). Inset barplot shows average slope of firing rate per current step (*P*=1). Data are represented as mean±s.e.m. Two-sided *t*-tests were used, unless stated otherwise.

Considering that we found a reduction of cerebellar AMPA receptor expression in global *Shank2*^−/−^ mice, we next examined neurotransmission at the PF–PC synapse using *ex vivo* whole-cell recordings (at 21±1 °C) (Fig. 2a). PF–PC EPSCs, which were obtained in WT and *Shank2*^−/−^ under comparable conditions (holding current: WT: −389±102 pA; *Shank2*^−/−^ −388±114 pA, *P*=1; PC input resistance: WT: 67.2±16.8 MΩ; *Shank2*^−/−^: 69.1±12.4 MΩ; *P*=0.8; Fig. 2b,c), revealed no significant differences in rise time (WT: 2.1±0.7 ms; *Shank2*^−/−^: 1.7±0.6 ms; *P*=0.2) or decay time (WT: 9.7±0.8 ms; *Shank2*^−/−^: 9.3±0.3 ms; *P*=0.3) (Fig. 2d,e). Moreover, evoking PF-EPSCs using stimulation currents varying from 3 to 15 μA resulted in similar event amplitudes (*P*=0.9, repeated-measures ANOVA) (Fig. 2f,g) and applying inter-stimulus intervals varying from 50 to 200 ms evoked comparable levels of paired-pulse facilitation (*P*=0.2, repeated-measures ANOVA) (Fig. 2h), indicating that baseline PF–PC synaptic transmission is unaltered by the lack of *Shank2*. Next, we evaluated whether the loss of *Shank2* affected neurotransmission at the climbing fibre (CF) to PC synapse. CF stimulation induced PC complex spikes in WT and *Shank2*^−/−^. These waveforms showed no significant differences in the amplitude of the initial Na^+^-spike (WT: 51.8±6.4 mV; *Shank2*^−/−^: 48.5±5.9 mV, *P*=0.3) and in the number of subsequent Ca^2+^-spikelets (WT: 1.6±0.5; *Shank2*^−/−^: 2.0±0.7; *P*=0.2) or the amplitude of Ca^2+^-spikelets (WT: 31.8±11.9 mV; *Shank2*^−/−^: 33.9±6.4 mV, *P*=0.7) (Supplementary Fig. 2a–c). Moreover, at P9–10 virtually all PCs of both WT and *Shank2*^−/−^ were innervated by multiple CFs, while at P25–35 all converted into mono-innervation (number of CF responses P9–10: WT: 2.0±0.5; *Shank2*^−/−^: 2.3±0.5; *P*=0.2; P25–35: WT: 1.0±0.0; *Shank2*^−/−^: 1.0±0.0; *P*=1, MWU-test) (Supplementary Fig. 2d,e). Finally, the characteristic paired-pulse depression of CF–PC synaptic transmission showed no differences throughout the tested developmental stages (P9–10: WT: 0.59±0.14; *Shank2*^−/−^: 0.54±0.11; *P*=0.5; P25–35: WT: 0.75±0.11; *Shank2*^−/−^: 0.77±0.1; *P*=0.6) (Supplementary Fig. 2f,g), together indicating that the CF to PC input in *Shank2*^−/−^ mice is not only normal in its baseline characteristics but also with respect to developmental elimination^31^.

To examine PC kinetics, we evoked action potentials (APs) using depolarizing current steps at near-physiological temperature (33±1 °C) (Fig. 2i). Evoked APs showed comparable thresholds (WT: −51.4±3.9 mV; *Shank2*^−/−^: −51.0±3.5 mV; *P*=0.8), amplitudes (WT: 39.8±5.8 mV; *Shank2*^−/−^: 35.9±5.8 mV; *P*=0.1) and half-widths (WT: 0.29±0.02 ms; *Shank2*^−/−^: 0.30±0.03 ms; *P*=0.7), as well as after-hyperpolarization amplitudes (WT: 6.5±1.6 mV; *Shank2*^−/−^: 7.5±1.7 mV; *P*=0.2) (Fig. 2j–m). In addition, PC intrinsic excitability was normal^32^ in that current step injections of increasing amplitude resulted in a linear current-to-firing frequency relationship (*P*=0.1, repeated-measures ANOVA) (Fig. 2n,o) with a similar slope (WT: 16.2±2.2 Hz; *Shank2*^−/−^: 16.1±2.3 Hz; *P*=1.0) (Fig. 2o). Together, these findings indicate that both the baseline transmission at PC excitatory synapses and PC intrinsic excitability remain intact in global *Shank2*^−/−^ mice.

### Increased sIPSCs and spiking irregularity in *Shank2* ^−/−^ PCs

**Figure 3:**
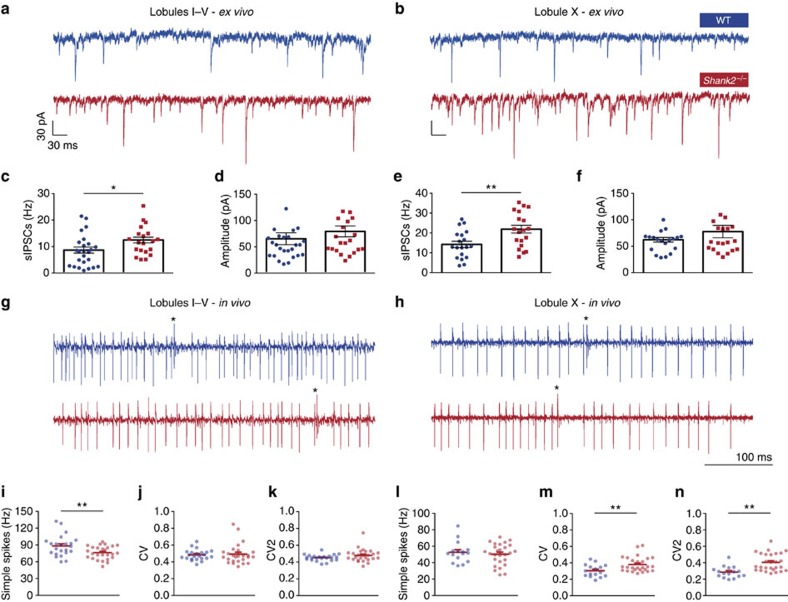
Increased spontaneous inhibitory events and higher simple spike irregularity in *Shank2*^−/−^ Purkinje cells. (**a**,**b**) Example of spontaneous firing inhibitory post synaptic currents (sIPSCs) in lobules I–V and X. (**c**,**e**) A higher frequency of sIPSCs is found in both anterior (I–V) (*P*=0.0295) and posterior (X) lobules (*P*=0.0079) in *Shank2*^−/−^ PCs (anterior: WT, *n*=25/3, cells per animals; *Shank2*^−/−^, *n*=20/3; posterior: WT, *n*=19/3; *Shank2*^−/−^, *n*=19/3). (**d**,**f**) There were no significant differences in sIPSC amplitudes anteriorly (*P*=0.1) or posteriorly (*P*=0.5). (**g**,**h**) Extracellular traces of PCs recorded in anterior (left) and posterior (right) lobules in the cerebellum, in WT and *Shank2*^−/−^. Asterisks denote complex spikes. (**i**–**k**) Simple spike (SS) firing frequency was significantly lower (*P*=0.0096) in *Shank2*^−/−^ (*n*=26/3) compared with wild type (*n*=23/3), whereas the coefficient of variation (CV) (*P*=0.7) and CV2 (*P*=0.1) did not differ. (**l**–**n**) In posterior lobule X, while SS firing frequency was similar (*P*=0.6), CV (*P*=0.0086) and CV2 (*P*=0.0003) were significantly higher in *Shank2*^−/−^ (*n*=27/3) compared with WT (*n*=16/3). Data are represented as mean±s.e.m. Single and double asterisks indicate *P*<0.05 and *P*<0.01, respectively. Two-sided *t*-tests were used, unless stated otherwise.

To investigate inhibition of PCs in global *Shank2*^−/−^ mice, we recorded spontaneous inhibitory postsynaptic currents (sIPSCs). Since PC activity can be related to the presence or absence of the glycolytic enzyme aldolase c (referred to as zebrin)^33^, we recorded from the predominantly zebrin-negative anterior lobules I–V, as well as the predominantly zebrin-positive posterior lobule X of the cerebellar cortex (Fig. 3a,b). In both regions, we observed an increase in the frequency (lobules I–V: WT: 8.3±5.9 Hz; *Shank2*^−/−^: 12.2±5.4 Hz; *P*=0.0295; lobule X: WT: 14.2±7.0 Hz; *Shank2*^−/−^: 21.5±8.8 Hz; *P*=0.0079; Fig. 3c,e), but not in the amplitude (for lobules I–V, WT: 53.2±24.4 pA; *Shank2*^−/−^: 65.2±29.5 pA; *P*=0.1; for lobule X, WT: 58.9±19.0 pA; *Shank2*^−/−^: 64.0±24.7 pA; *P*=0.5) of sIPSCs (Fig. 3d,f). Importantly, *Shank2*^−/−^ sIPSC frequency was higher in lobule X than in the anterior lobe (*P*=0.0002). Given that inhibition decreases the firing frequency of PCs, but increases their irregularity^34^,^35^, we hypothesized that the increased frequency of sIPSCs in *Shank2*^−/−^ would translate into an overall decrease of *in vivo* SS activity but with an increased irregularity (Fig. 3g,h). The global *Shank2*^−/−^ mice did indeed exhibit a decrease in firing frequency in lobules I–V (WT: 88.2±18.7 Hz; *Shank2*^−/−^: 76.3±11.8 Hz; *P*=0.0096) (Fig. 3i), but notably not in lobule X (WT: 52.6±12.7 Hz; *Shank2*^−/−^: 50.3±12.9 Hz; *P*=0.6) (Fig. 3l). Conversely, and consistent with the relative magnitude of the change in sIPSC frequency, the irregularity of PC SS firing was increased in lobule X (CV: WT, 0.30±0.08; *Shank2*^−/−^, 0.38±0.09, *P*=0.0086; CV2: WT, 0.29±0.07; *Shank2*^−/−^, 0.40±0.11, *P*=0.0003), but not in lobules I–V (CV: WT, 0.48±0.06; *Shank2*^−/−^, 0.49±0.12, *P*=0.7; CV2: WT, 0.45±0.04; *Shank2*^−/−^, 0.48±0.08, *P*=0.1) (Fig. 3j–n). The complex spike frequency and the pause in SS firing following each complex spike was similar between global *Shank2*^−/−^ mice and their WT littermates in both the anterior (WT: frequency 1.28±0.29 Hz; pause 9.11±1.98 ms; *Shank2*^−/−^: 1.32±0.24 Hz; 10±2.9 ms; *P*=0.7 and *P*=0.2, respectively) and posterior lobules (WT: frequency 0.67±0.19 Hz; pause 19.7±5.5 ms; *Shank2*^−/−^: frequency 0.84±0.37 Hz; pause 17.61±5.29 ms; *P*=0.1 and *P*=0.2, respectively) (Supplementary Fig. 3). Thus, in the absence of *Shank2*, the zebrin-positive lobule X selectively exhibits a highly irregular pattern of PC SS firing, which is consistent with a relative increase of inhibitory input onto lobule X PCs.

### Impaired synaptic and intrinsic plasticity in *Shank2* ^−/−^ PCs

**Figure 4:**
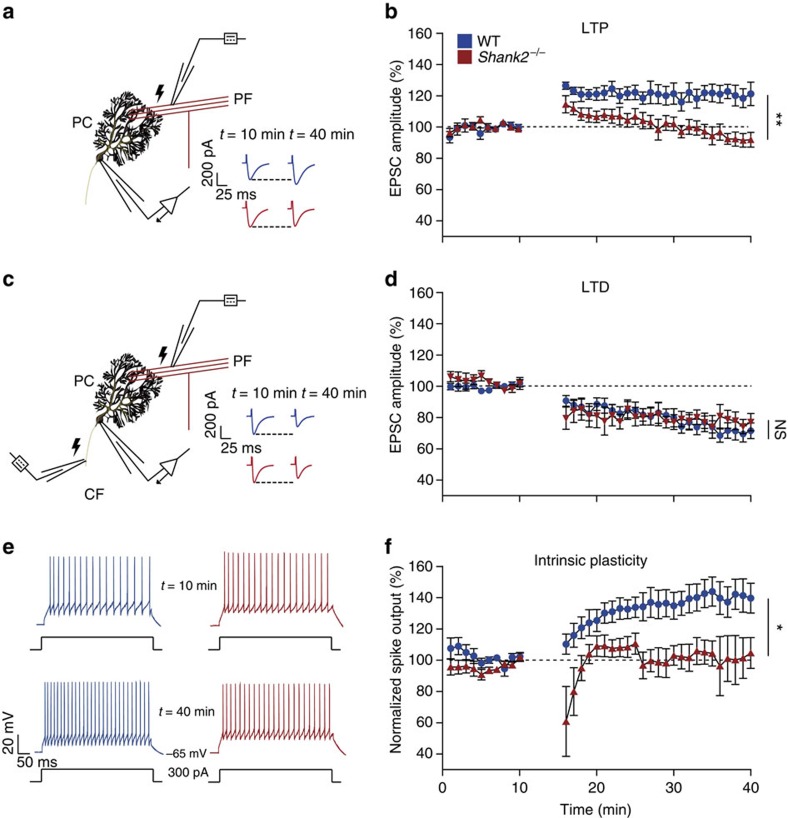
Impaired synaptic and intrinsic plasticity in *Shank2*^−/−^ *ex vivo*. (**a**) Recording configuration for PF-LTP experiments. Inset: example of five averaged EPSCs for WT (blue) and *Shank2*^−/−^ (red) before LTP induction (at 10 min) and after LTP induction (at 40 min). (**b**) LTP experiment with 5 min PF stimulation at 1 Hz inducing LTP in WTs (*n*=7/6, cells per animals, *P*=0.0027) but not in *Shank2*^−/−^ PCs (*n*=12/6, *P*=0.3), which is reflected in the difference between genotypes (*P*=0.0066). (**c**) Recording configuration for PF-LTD experiments. Inset: example traces as in **a**. (**d**) LTD is induced in both WT (*n*=9/6, *P*<0.0001) and *Shank2*^−/−^ (*n*=7/6, *P*=0.0009) PCs, to a similar degree (*P*=1). (**e**) Example of traces for intrinsic plasticity with current injections of 300 pA. (**f**) LTP induction protocol induced enhanced spike output in WT PCs (*n*=5/4, *P*=0.0053), but not in *Shank2*^−/−^ PCs (*n*=5/4, *P*=0.5), as reflected in their difference (*P*=0.0201). Data are represented as mean±s.e.m. Single and double asterisks indicate *P*<0.05 and *P*<0.01, respectively. All tests were repeated-measures ANOVAs. NS, not significant.

Given that *Shank2* functions as a PSD scaffolding protein of postsynaptic receptors^17^,^30^ for which we observed decreased expression of both GluA1 and GluA2 in cerebellar synaptosomes of *Shank2*^−/−^ mice (Fig. 1), we reasoned that PC synaptic plasticity might also be affected^25^,^26^. Induction of long-term potentiation (LTP) (21±1 °C; 1 Hz, 5 min PF-tetanus) (Fig. 4a) reliably increased PF-EPSC amplitudes in WT PCs (121.1±19.8% at *t*=40 min; *P*=0.003, repeated-measures analysis of variance (ANOVA)), but not in those of global *Shank2*^−/−^ mice (91.8±14.2% at *t*=40; *P*=0.3, repeated-measures ANOVA) (Fig. 4b). In contrast, both WT and global *Shank2*^−/−^ mice exhibited robust long-term depression (LTD) of PF-EPSCs following co-activation (33±1 °C; 1 Hz) of PFs and CFs (WT: 71.4±14.9%; *P*<0.0001; *Shank2*^−/−^: 76.9±14.6%; *P*=0.0009, repeated-measures ANOVA) (Fig. 4c,d). Since LTP has been reported to facilitate adaptation of intrinsic properties, driving spike activity^32^, we next examined PC intrinsic plasticity before and after PF-LTP induction (Fig. 4e). While WT mice readily demonstrated a potentiation of intrinsic excitability (139.7±21.3% at *t*=40 min; *P*=0.005, repeated-measures ANOVA), intrinsic plasticity was markedly impaired in global *Shank2*^−/−^ mice (104.6±22.2% at *t*=40 min; *P*=0.5, repeated-measures ANOVA) (Fig. 4f). These results suggest that *Shank2* is a critical modulator of both synaptic and intrinsic plasticity in PCs.

### Expression of *Shank2* in *L7-Shank2* ^−/−^ mice

**Figure 5:**
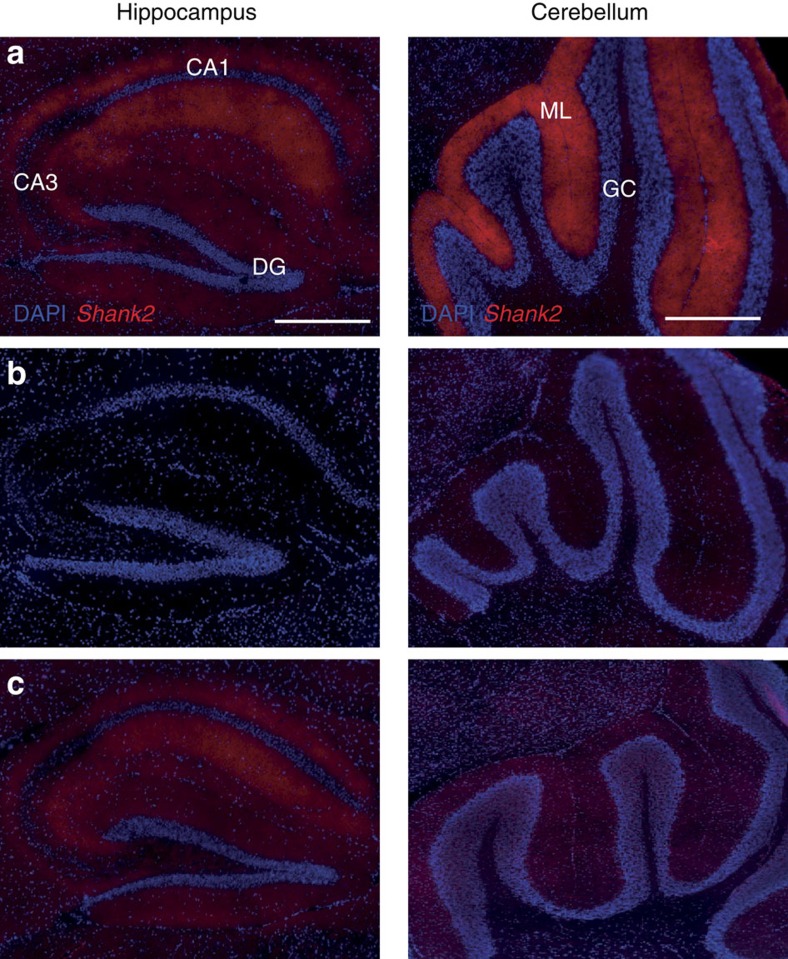
Immunohistological staining of the *Shank2* protein in *Shank2*^−/−^ and *L7-Shank2*^−/−^ hippocampus and cerebellum. (**a**) Sagittal cryosection of hippocampal SHANK2 (red) and nucleus staining (DAPI; blue) in a WT (left) *L7-Shank2*^−/−^. Scale bar, 500 μm. Sagittal cryosection of cerebellar SHANK2 staining (right). Scale bar, 200 μm. (**b**) Staining for SHANK2 in the hippocampus and cerebellum of the global *Shank2*^−/−^ shows absence of expression. (**c**) The *L7-Shank2*^−/−^ shows expression in the hippocampus, but not in the molecular layer of the cerebellum.

To explore the behavioural impact of the lack of *Shank2* in PCs, we generated a PC-specific KO of *Shank2 (*see Methods section) using the floxed version of the *Shank2*^−/−^ mutants^25^ and the L7-vector^32^. Immunocytochemical analysis with the SA5193 rabbit primary SHANK2 antibody^25^,^36^ confirmed that SHANK2 was specifically deleted in PCs in the PC-specific *L7-Shank2*^−/−^ mice, but not in WT littermates, whereas it was ubiquitously deleted in the global *Shank2*^−/−^ (Fig. 5). Importantly, PC-specific deletion of *Shank2* had no discernible impact on cellular zebrin identity or on the zonal patterns of zebrin staining across cerebellar modules (Supplementary Fig. 4).

### Impaired motor learning in *L7-Shank2* ^−/−^ mice

Given the variety of electrophysiological aberrations in PCs in the global *Shank2*^−/−^, we next examined motor behaviour in the PC-specific *L7-Shank2*^−/−^ mice. Unlike the hyperactivity exhibited by global *Shank2*^−/−^ mice in an open field^25^,^26^, mice with PC-specific deletion of *Shank2* exhibited no evidence of hyperactivity in the open field test compared with their WT littermates (velocity: WT: 12.28±2.79 cm s^−1^; *L7-Shank2*^−/−^: 13.24±2.61 cm s^−1^; *P*=0.3; distance moved: WT: 7.37±1.68 m; *L7-Shank2*^−/−^: 7.94±1.57 m; *P*=0.3) (Supplementary Fig. 5a,b). The lack of hyperactivity was confirmed using the PhenoTyper Box (Noldus), in which free exploration was quantified over a 30 min period in a homecage-like environment (velocity: WT: 6.7±1.1 cm s^−1^; *L7-Shank2*^−/−^: 6.3±1.3 cm s^−1^; *P*=0.5, distance moved: WT: 384.6±68.8 cm; *L7-Shank2*^−/−^: 371.7±79.7 cm; *P*=0.5, repeated-measures ANOVA) (Supplementary Fig. 5c,d). Moreover, during the ErasmusLadder test^37^ motor performance was similar between genotypes, including the efficiency and timing of steps (second day efficiency, WT: 33.1±20.0%; *L7-Shank2*^−/−^: 47.1±15.4 cm; *P*=0.3; second day timing: WT: 359.2±84.2 ms; *L7-Shank2*^−/−^: 330.7±49.4 ms; *P*=0.8, repeated-measures ANOVA) (Supplementary Fig. 5e,f). Finally, the amplitude (gain) and timing (phase) of baseline optokinetic (OKR) (OKR gain, *P*=0.6, OKR phase, *P*=0.9, repeated-measure ANOVA) and vestibulo-ocular reflexes (VOR) (VOR gain, *P*=0.4, VOR phase, *P*=0.2, repeated-measure ANOVA) were also similar (Supplementary Fig. 5g–j), further highlighting that motor performance is normal in *L7-Shank2*^−/−^ mutants.

**Figure 6:**
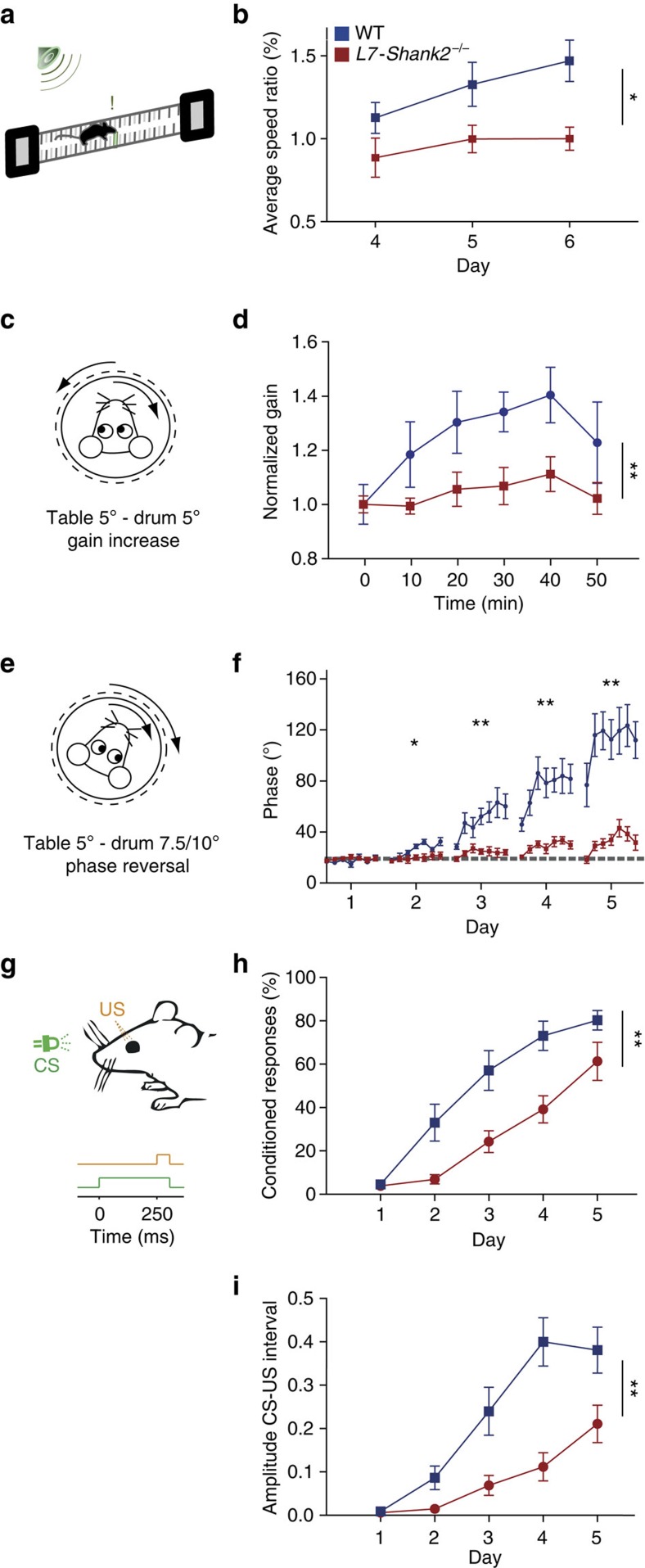
*L7-Shank2*^−/−^ mice show impaired motor learning. (**a**,**b**) After 3 days of training, WT (*n*=9) but not *L7-Shank2*^−/−^ mice (*n*=6) (*P*=0.018) learned to increase their speed during a conditioned ErasmusLadder test using tone-cued rung displacements. (**c**,**d**) In vestibulo-ocular reflex (VOR) gain increase training, *L7-Shank2*^−/−^ mice (*n*=8) were not able to adapt their gain like WTs did (*n*=7) (*P*=0.006). (**e**,**f**) *L7-Shank2*^−/−^ mutants (*n*=9) did not adapt their VOR phase following a reversal training paradigm, whereas WT (*n*=9) did (second day, *P*=0.047; third, *P*=0.0013; fourth, *P*<0.0001; fifth, *P*=0.0003). (**g**–**i**) Impaired percentage (*P*=0.0013) and amplitude (*P*=0.0009) of conditioned responses (CRs) in *L7-Shank2*^−/−^ mice (*n*=10) compared with WT (*n*=11) in an eye-blink conditioning paradigm (200 paired trials daily). Data are represented as mean±s.e.m.. Single and double asterisks indicate *P*<0.05 and *P*<0.01, respectively. All tests were repeated-measures ANOVAs.

In contrast, motor learning was consistently affected in a variety of cerebellar motor learning tasks (Fig. 6). Using a conditioning task within the ErasmusLadder, in which mice were presented with a tone preceding the elevation of an obstructive rung at a 200 ms interval^37^, *L7-Shank2*^−/−^ mice were unable to successfully avoid the obstacle (*L7-Shank2*^−/−^ versus WT: *P*=0.018, repeated-measures ANOVA) (Fig. 6a,b). Furthermore, *L7-Shank2*^−/−^ mice failed to acquire the normal increase in VOR gain (*L7-Shank2*^−/−^ versus WT: *P*=0.006, repeated-measures ANOVA) or shift in VOR phase (second day; *P*=0.047, third *P*=0.0013, fourth *P*<0.0001, fifth *P*=0.0003, repeated-measures ANOVA) following visuovestibular mismatch training^38^ (Fig. 6c–f). Finally, *L7-Shank2*^−/−^ mice exhibited a significant impairment of Pavlovian eye-blink conditioning^39^ using a light pulse as the conditioning stimulus (CS) and a corneal air puff as the unconditioned stimulus at a 250 ms interval (conditioned response or corneal reflection (CR) rate: *P*=0.0013; CR amplitude: *P*=0.0009; repeated-measures ANOVA; Fig. 6g–i). These findings indicate that *L7-Shank2*^−/−^ mice have normal baseline motor performance, but prominent impairments in motor learning.

### Irregular *in vivo* PC SS in *L7-Shank2* ^−/−^ mice

**Figure 7:**
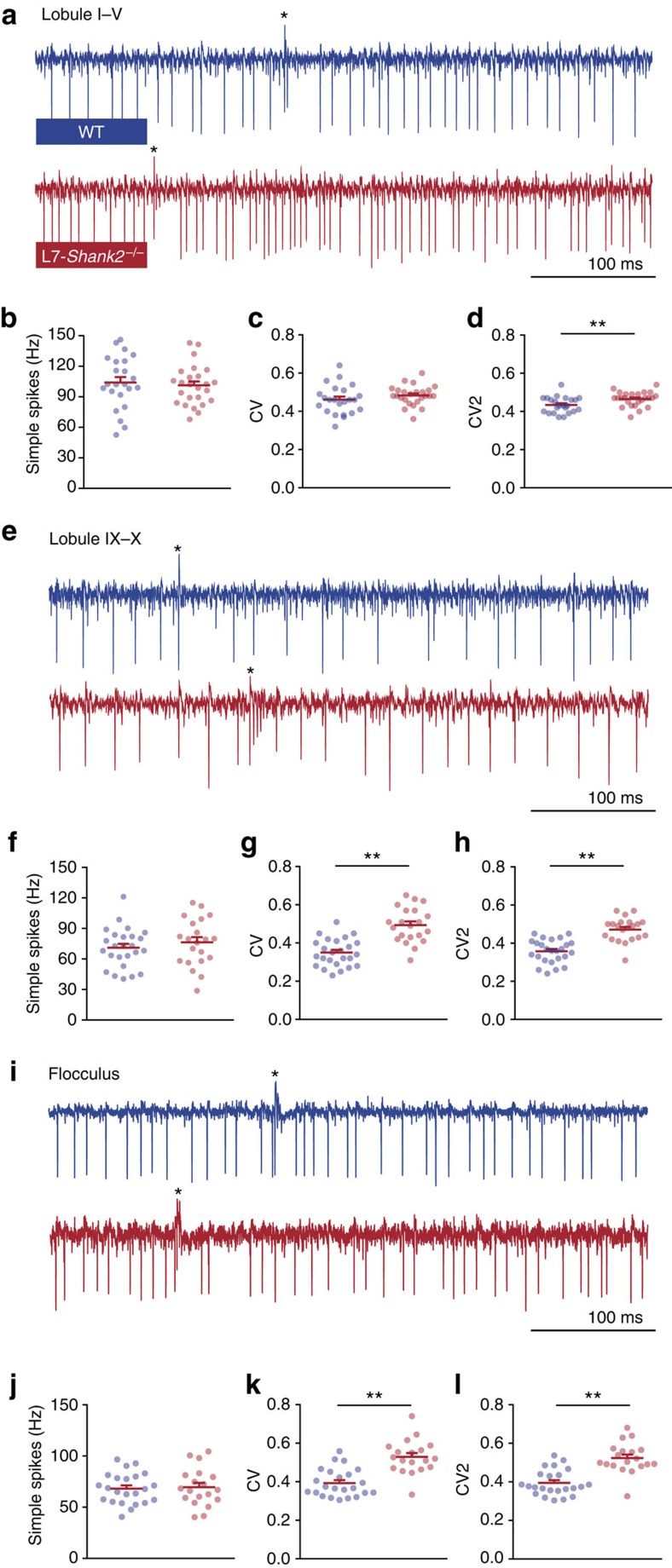
*In vivo* simple spike firing characteristics in *L7-Shank2*^−/−^ Purkinje cells. (**a**) Extracellular PC traces recorded from anterior lobules (I–V) in WT (top) and *L7-Shank2*^−/−^ (bottom) mice. (**b**–**d**) Firing characteristics in the anterior lobules reveal a difference in CV2 (*P*=0.0092) between *L7-Shank2*^−/−^ (*n*=25/3, cells per animals) and WT (*n*=23/3). (**e**) Example PC traces from posterior lobules (IX–X). (**f**–**h**) PCs from the posterior cerebellum in *L7-Shank2*^−/−^ (*n*=21/3) showed significantly higher CV (*P*<0.0001) and CV2 (*P*<0.0001) values compared with WT (*n*=25/3). (**i**) Example PC traces from posteriorly located flocculus, which is responsible for VOR learning. (**j**–**l**) Again, *L7-Shank2*^−/−^ (*n*=19/2) shows significantly higher CV (*P*<0.0001) and CV2 (*P*<0.0001) values than WT (*n*=23/2). Double asterisks denote *P*<0.01. All tests were two-sided *t*-tests.

To investigate whether the changes in electrophysiological properties observed in PCs of the global *Shank2*^−/−^ mice may contribute to the behavioural phenotypes observed, we tested to what extent the changes in SS activity also occurred in the *L7-Shank2*^−/−^ mice. We first recorded extracellular single units *in vivo* from the largely zebrin-negative lobules I–V (Fig. 7a–d) and the predominantly zebrin-positive lobules IX–X (Fig. 7e–h). Importantly, the recordings in the *L7-Shank2*^−/−^ mice fully reproduced the increases in CV (WT, 0.35±0.07; *L7-Shank2*^−/−^, 0.49±0.09; *P*<0.0001) and CV2 (WT, 0.36±0.06; *L7-Shank2*^−/−^, 0.47±0.06; *P*<0.0001) (Fig. 7g,h) that were found in the posterior lobules of the global *Shank2*^−/−^ (Fig. 3i–n), confirming the higher SS irregularity. In addition, the *L7-Shank2*^−/−^ mice also showed signs of SS irregularity in the anterior lobules in that their CV2 was also significantly increased (WT, 0.43±0.04; *L7-Shank2*^−/−^, 0.47±0.04; *P*=0.0092) (Fig. 7c,d). The *L7-Shank2*^−/−^ SS mice activity did not show higher firing frequencies in either the anterior (WT: 104.4±25.8 Hz; *L7-Shank2*^−/−^: 101.2±19.7 Hz; *P*=0.6) or posterior (WT: 70.9±19.5 Hz; *L7-Shank2*^−/−^: 76.3±23.4 Hz; *P*=0.4) lobules (Fig. 7b,f). Finally, we also recorded SS activity of PCs in the flocculus of the vestibulocerebellum, because they are known to directly control VOR adaptation^34^ (Fig. 7i–l). In PCs that were identified to be related to VOR adaptation by their response to motion around the vertical axis in space, we again found a significant increase in SS irregularity (CV: WT, 0.39±0.07; *L7-Shank2*^−/−^, 0.53±0.08; *P*<0.0001; CV2: WT, 0.39±0.07; *L7-Shank2*^−/−^, 0.52±0.08; *P*<0.0001), while their overall firing frequency was unaffected (WT: 68.2±15.0 Hz; *L7-Shank2*^−/−^: 69.6±18.8 Hz; *P*=0.8) (Fig. 7j–l). Moreover, *L7-Shank2*^−/−^ showed a bigger difference with WT in SS irregularity in the posterior lobules (40.0% and 30.6% higher CV and CV2, respectively) compared with that in the anterior lobules (4.4% and 9.3% higher CV and CV2, respectively). No significant differences were observed in the duration of the complex spike-induced SS pause or the complex spike firing frequency in any of the recorded lobules (*P*>0.2 in all cases) (Supplementary Fig. 6). Together, *L7-Shank2*^−/−^ mice demonstrate the critical importance of *Shank2* in PCs for maintaining SS regularity.

### Abnormal social and repetitive behaviour in *L7-Shank2* ^−*/*−^ mice

**Figure 8:**
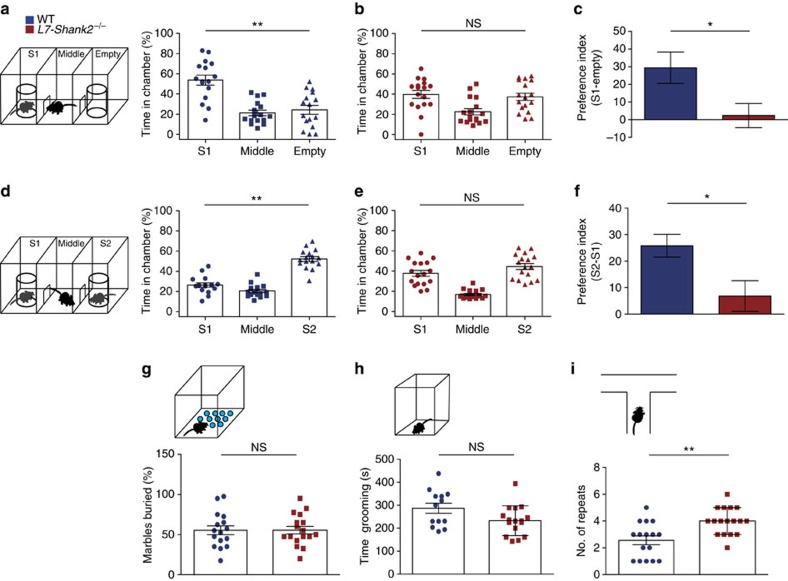
*L7-Shank2*^−/−^ mice show social impairment and signs of task-specific repetitive behaviour. (**a**) Three-chamber social interaction evaluated by relative time spent in each chamber. WTs (*n*=16) prefer to spend time in the room with the stranger 1 mouse (S1), compared with the empty room (*P*=0.0002, MWU-test). (**b**) This was not the case for *L7-Shank2*^−/−^ (*n*=17) mice (*P*=0.7, MWU-test). (**c**) The preference index (S1-empty) confirms the difference between genotypes (*P*=0.021). (**d**) Following the introduction of a second stranger (S2), WTs (*n*=16) prefer to spend time in the chamber with S2 compared with that with S1 (*P*=0.0001, MWU-test). (**e**) The *L7-Shank2*^−/−^ mice (*n*=17) did not show a preference for newly introduced S2 (*P*=0.1, MWU-test). (**f**) The S1−S2 preference index indicates that WTs prefer S2 more than *L7-Shank2*^−/−^ do (*P*=0.013). (**g**) No difference was found in a marble-burying task indicative of anxious and/or repetitive behaviour (WT, *n*=16; *L7-Shank2*^−/−^, *n*=17, *P*=1.0). (**h**) *L7-Shank2*^−/−^ (*n*=16) seemed to trend towards less grooming than WTs (*n*=13) (*P*=0.054). (**i**) T-maze paradigm showed less consecutive alternations in *L7-Shank2*^−/−^ (*n*=17) compared with WT (*n*=16) (*P*=0.0023, MWU-test) indicating repetitive decision-making. Data are presented as mean±s.e.m. Single and double asterisks indicate *P*<0.05 and *P*<0.01, respectively. Two-sided *t*-tests were used, unless stated otherwise. NS, not significant.

We next examined social and repetitive ASD-like behaviours in the PC-specific *L7-Shank2*^−/−^ mice. The three-chamber social interaction task is a widely used social interaction paradigm for evaluating ASD-like behaviour in mouse models of autism^25^,^26^. WT mice exhibited a normal preference for the chamber in which the stranger mouse (S1) was present, compared with the empty chamber (*P*=0.0002, MWU-test) (Fig. 8a). In contrast, *L7-Shank2*^−/−^ mice had no preference for either S1 or the empty chamber (*P*=0.7, MWU-test) (Fig. 8b). Comparing the preference index (stranger-empty) between WT and *L7-Shank2*^−/−^ mice revealed a significantly decreased preference of *L7-Shank2*^−/−^ mice for the stranger mouse (*P*=0.0213) (Fig. 8c), indicating their social interaction deficits. With the introduction of a second stranger in the previously empty chamber, WT mice again demonstrated an increased preference for the novel stranger (S2), this time compared with the familiar mouse (S1) (*P*=0.0001, MWU-test) (Fig. 8d), whereas *L7-Shank2*^−/−^ mice showed no preference for either the familiar or the novel stranger mice (*P*=0.1, MWU-test) (Fig. 8e). Comparing the preference index (S2−S1) between WT and *L7-Shank2*^−/−^ confirmed the impairment of social interaction in *L7-Shank2*^−/−^ mice (*P*=0.0136) (Fig. 8f). Because of previously reported compulsive grooming^25^ and jumping^26^ in global *Shank2*^−/−^ mice, we next examined repetitive behaviour. We observed no significant differences between WT and *L7-Shank2*^−/−^ mice in the percentage of buried marbles in the marble-burying task (*P*=1.0) (Fig. 8g) or in the duration of grooming over a 15 min period (*P*=0.054) (Fig. 8h). However, the *L7-Shank2*^−/−^ mice did show an increase of repetitive behaviour in the T-maze through an increased perseveration (*P*=0.0023, MWU-test) (Fig. 8i). Finally, we observed no significant difference in anxiety (*P*=0.7, *χ*^2^-test) or olfactory sensitivity (*P*=0.6) of *L7-Shank2*^−/−^ mice that could have potentially biased the social behaviour assessments (Supplementary Fig. 7). Together, these results indicate that *L7-Shank2*^−/−^ mice exhibit ASD-like social impairments and task-specific repetitive behaviour.

## Discussion

Severe loss-of-function mutations in *SHANK2* have been firmly established as conferring a high genetic risk for ASD and intellectual disability^20^,^22^,^29^. ASD patients with disruptive *SHANK2* mutations exhibit motor impairments, language delay and cerebellar dysfunction including dysmetria and dysdiadochokinesis^29^. Considering the increasing evidence for cerebellar involvement in ASD^40^, we investigated anatomical, molecular and physiological consequences of global *Shank2* ablation in the cerebellum. In addition, we analysed *L7-Shank2*^−/−^ mice with cerebellar PC-specific deletion of *Shank2* to evaluate the extent to which the ASD-related behavioural findings in global *Shank2*^−/−^ mice can be attributed to cerebellar dysfunction.

In recent years, several genetic mouse models for ASD have been used for the study of cerebellar abnormalities. The first study to implement a PC-specific model related to ASD involved the deletion of *Fmr1*, the gene encoding the fragile X mental retardation 1 protein^41^. In this study, the authors reported eye-blink abnormalities and increased LTD in both global and PC-specific deletion of *Fmr1*. Furthermore, global *Nlgn3*-KO mutants exhibited deficits in cerebellum-related motor performance as assessed by the ErasmusLadder^42^. A more recent model examining the 15q11-13 duplication ASD syndrome demonstrated impaired cerebellar synaptic plasticity and motor learning deficits as assessed by eye-blink conditioning^43^. Perhaps the most definitive study implicating cerebellar dysfunction as aetiologic for ASD-like behaviour involved a PC-specific deletion of *Tsc1* (ref. 44). This study was the first to demonstrate that PC-specific deletion of an ASD-associated gene results in ASD-like behaviour. Finally, a very recent study using multiple mouse models of syndromic ASD found a consistent pattern of impaired sensorimotor integration^45^. These studies have established the foundation by which the cerebellar synaptic pathophysiology underlying ASD can be mechanistically investigated^18^.

Given previous studies reported on the importance of SHANK2 in the regulation of neuronal plasticity^25^,^26^, we investigated both synaptic and intrinsic plasticity of cerebellar PCs (Fig. 4). Our results indicate that SHANK2 is crucial for PF–PC LTP, but not LTD. In addition, we show that SHANK2 is important for intrinsic plasticity of neuronal excitability^46^. In contrast to a recent study of the 15q11-13 duplication ASD syndrome in which PC synaptic plasticity deficits were limited to LTD^43^, our results now highlight LTP impairments as a candidate mechanism underlying the cerebellar pathophysiology of ASD. SHANK2 is a dedicated scaffolding protein, which has a major role in the regulation of glutamate receptor integration, synaptic transmission and plasticity^17^,^47^. Future molecular and functional studies will have to elucidate the exact mechanisms by which SHANK2 mediates plasticity in the PC, but it may well include a suboptimal integration of GluR subunits as the expression levels of GluA1 and GluA2 were both reduced in the *Shank2*^−/−^ mice. Since GluR subunit levels were analysed in synaptosomes from whole adult cerebella, it remains to be investigated to what extent these reductions result from specific changes at the PF to PC synapse and/or other cerebellar cortical synapses, and at what developmental stage they start to occur.

Because of the cerebellar physiological impairments and the previously reported motor hyperactivity in *Shank2*^−/−^ mice^25^,^26^, we examined activity levels during both baseline exploration and motor learning. To our surprise, we did not find motor performance abnormalities in the PC-specific *L7-Shank2*^−/−^ mice during various assessments including five separate locomotion and eye movement tests (Supplementary Fig. 2). However, we did observe substantial impairments of cerebellar motor learning including conditioning of locomotion and eye-blink responses, as well as adaptation of compensatory eye movements (Fig. 6). It might seem counterintuitive that baseline motor performance can be intact while the capacity for motor learning is reduced, but this combination has been observed in many different mutant lines over the last few decades^48^. Most likely, it reflects the indispensable role of PC plasticity for the acquisition of new behaviours within relatively short periods of time as occurs during the experimental training paradigms (that is, in the order of hours), and the ability of the motor performance control system to compensate upstream and/or downstream of the affected synapse when prolonged adaptation periods are available as occurs during postnatal development (that is, in the order of weeks)^49^. The potential causality of the identified electrophysiological abnormalities as underlying the motor learning impairments is strengthened by our independent findings in another PC-specific mouse mutant (*L7-PP2B*^−/−^), in which also both synaptic LTP and intrinsic plasticity were affected^32^. Together, these phenotypes point towards a PC-dependent contribution to the behavioural motor impairments frequently observed in ASD.

In addition to changes in plasticity, we also found that inhibition of PCs was enhanced in the global *Shank2*^−/−^ mutants. Since reduced inhibition of PCs increases regularity of SS activity^34^, we hypothesized that PCs of the global *Shank2*^−/−^ mutants should have a higher level of irregularity of SS firing (Fig. 3). Indeed, this hypothesis was not only consistent with the *in vivo* extracellular recordings in vermal lobule X of the global *Shank2*^−/−^, but also confirmed in three different areas (Lobules I–V, Lobules IX–X and the flocculus) of the *L7-Shank2*^−/−^ mice (Fig. 7). Moreover, this correlation was also in line with the fact that the differences in sIPSCs, CV and CV2 had bigger effect sizes in the posterior lobules than the anterior lobules.

The increased frequency of inhibition in the global *Shank2*^−/−^ did not occur concomitantly with increased amplitude of sIPSCs postsynaptically at PCs, indicating that the observed effect could be of pre-synaptic origin. The PC irregularity in the *L7-Shank2*^−/−^ would then have to originate from a pre-synaptic effect of the postsynaptic absence of SHANK2. Indeed, recent evidence indicates the possibility of Shank-mediated transsynaptic signalling through transmembrane proteins affecting both post and pre-synaptic processes important for vesicle release probability^50^. This type of transsynaptic signalling could manipulate the inhibitory input to PCs either directly or indirectly, for example, through altered glutamate spillover from the CF to PC synapse^51^. Future research aimed at pinpointing the sites relevant to the effects described above should thus focus not only on PC-specific mouse mutants, but also on those in which their afferents are specifically affected^52^,^53^. In doing so, important consideration in studies implementing cell-specific deletions should be given to germ-line analyses, given the sensitivity of the *L7*-cre^44^,^54^ and *Shank2* lines (Supplementary Table 1)^55^,^56^.

We observed a significant decrease in SS frequency in the anterior lobules of global *Shank2*^−/−^ mice, but not in their posterior lobules, nor in the anterior or posterior lobules of *L7-Shank2*^−/−^ mice. We believe that this inconsistency may reflect the fact that the spontaneous SS firing frequency of PCs is probably largely due to their intrinsic properties rather than the synaptic efficacy of their inhibitory or excitatory inputs^33^. Indeed, blocking inhibitory or excitatory synaptic input to PCs by deleting their GABA-A-gamma2 receptor-subunits or abolishing voltage gated calcium channels at their PF input primarily affects the regularity of SS firing, rather than their firing frequency^34^,^53^. Thus, the consistent irregularity of SS in PCs, particularly in the posterior lobe, of the *Shank2*^−/−^ mutants underlines the putative importance of precise SS regularity for behavioural output^48^. Although abnormalities in the anterior and posterior lobules have both been proposed as relevant sites of cerebellar pathology in ASD^57^, our converging data obtained in the posterior lobe suggest that the mechanisms governing the regularity of SS firing reveal a common biological vulnerability in the aetiology of ASD.

Here we report impaired social and task-specific repetitive behaviour due to the PC-specific deletion of *Shank2* (Fig. 8). This result is particularly interesting as, to our knowledge, it is the first PC-specific mouse model for a non-syndromic form of autism in which ASD-like behaviour has been established. The impaired social behaviour, late-onset ataxia and reduced excitability of PCs previously observed in *L7-Tsc1* mice^44^ were due to the absence of a protein that inhibits mTOR signalling through which the translation of a wide variety of proteins is regulated. In contrast, here we show that disruption of the synapse through the absence of a single postsynaptic scaffolding protein in the PC is sufficient to show impaired ASD-related motor learning and social behavioural impairments. In addition to the social impairments, we found signs of enhanced repetitive behaviour in the T-maze paradigm, but not the marble-burying task or grooming tasks. Since the T-maze task reveals the level of cognitive inflexibility following decision-making over consecutive trials rather than the level of repetitious behaviour dominated by high-frequency motor activity that characterizes the other two tasks and that may well be confounded by deficits in cerebellar motor learning, these results highlight the importance of the PC synaptic function for ASD beyond the classically ascribed motor-related behaviour.

One of the main challenges remaining is to mechanistically explain the contribution of impaired PC physiology to the observed ASD behavioural phenotypes. As previously mentioned, the Shank family of postsynaptic scaffolding proteins has many different interacting proteins in the PSD through which they could contribute to the functional establishment of regulatory mechanisms for plasticity. The translational challenge from synapse to behaviour brings about two main questions: How does an impaired PC mediate ASD-related behaviour? And how might PC impairments lead to abnormal brain function beyond the cerebellum with regard to neurodevelopmental critical periods? The first question has been extensively addressed by the accumulating evidence regarding the contribution of ASD-related cerebellar dysfunction to impaired motor learning, as apparent from the eye movement adaptation, ErasmusLadder and eye-blink conditioning findings examined here and by other investigators^45^. It is indeed possible that the increased inhibition and irregularity of SS firing, in addition to impaired cerebellar plasticity mechanisms, may contribute to social and repetitive behaviour-related phenotypes in ASD. We believe that the answer to how the cerebellum can essentially contribute to socially impaired behaviour could reside in various mechanisms. The idea that disruption of a certain brain area during development could affect the development and consequently the function of other inter-connected areas, also termed developmental diaschisis, has recently been put forward as a prime mechanism for the cerebellum in its ability to influence other cortical areas in critical developmental periods^58^. In the future, the latter hypothesis can, for example, be tested with PC-specific *Shank2* ablation at different stages during development using inducible mouse models, as has recently been employed for other ASD-related genes^24^,^59^. These experiments will help to further elucidate the mechanisms by which differential genes, such as *Shank2*, regulate cerebellar function and ultimately ASD-like behaviour.

## Methods

Experiments and analyses were performed with the experimenters blinded to the genotype. Mice used were global germ-line *Shank2*^−/−^ and their littermate WTs all bred on a mixed C57BL6/N and C57BL6/J background. The generation of these mice has previously been described in detail^25^,^26^. The *L7-Shank2*^−/−^ was generated by crossing Purkinje specific L7(Pcp2)-Cre^54^ with *Shank2*^flxd/flxd^ (ref. 25). Genotyping was performed on postnatal day (P)7–10 using primers 1700 S (5′-TCCATGGTT TCGCGAGAGCG-3′), 1842 AS (5′-TCCCTATTGGGACGCAGTGG-3′) and 2394 AS (5′-CAGCATCATGACAATGTCTCCA-3′). For all experiments, we used mice from both genders, unless indicated otherwise. The mice were individually housed with food and water available ad libitum and in 12:12 h light/dark cycles. All experiments were approved by local and national ethical committees.

### Primary antibodies

The anti-SHANK2 SA5193 antibody has been characterized previously^25^. The following antibodies were from commercial suppliers: anti-GluA1 (Cat. No. 182 003), anti-GluA2 (Cat. No. 182 103), anti-Nlgn3 (Cat. No. 129 113) (all Synaptic Systems, Goettingen, Germany), anti-β3-Tubulin (Cat. No. MRB-435P) (Covance, Brussels, Belgium) and Aldolase C (Cat. No. 12065) (Santa Cruz, Dallas, USA)

### Golgi stainings

Adult mouse cerebella were dissected and prepared using the FD Rapid GolgiStain Kit (NeuroTechnologies, Vilnius, Lithuania). Serial coronal sections of 150 μm were collected from WTs and global *Shank2*^−/−^ mice and Z-stack images were taken using an upright Axioscope (Carl Zeiss, Jena, Germany). Distal dendrites of PCs were traced for spine analysis.

### Electron microscopy

Adult mice were transcardially perfused with fixative (2% paraformaldehyde, 2.5% glutaraldehyde, 1% saccharose in 0.1 M cacodylate buffer, pH 7.3) and their cerebella were dissected and post-fixed overnight at 4 °C. After dehydration and staining with 2% uranyl acetate, the material was embedded in epoxy resin. Ultrathin sections were cut using an ultramicrotome (Ultracut UCT, Leica). After lead citrate staining, sections from WTs and global *Shank2*^−/−^ mice were examined using an electron microscope (JEM 1400 TEM, Jeol). For ultrastructural PSD analysis, spine synapses have been selected in the distal molecular layer where the PF–PC contacts greatly outnumber other types of synapses.

### Biochemistry

Adult mouse cerebella were homogenized on ice in HEPES-buffered sucrose (320 mM sucrose, 5 mM HEPES, pH 7.4) containing protease inhibitor mixture (Roche, Mannheim, Germany). The homogenate was centrifuged (1,000*g*, 4 °C) to remove cell debris and nuclei. The supernatant was further centrifuged (12,000*g*, 4 °C) to obtain a pellet containing the cerebellar synaptosomes. Equal amounts of 10–20 μg protein per lane were separated by SDS–polyacrylamide gel electrophoresis and blotted onto polyvinylidene fluoride membranes using standard protocols. After incubation with primary antibodies (1:1,000 for anti-Shank1, anti-GluA1, anti-GluA2, anti-Nlgn3; 1:10,000 for anti-β3-Tubulin), immunoreactivity was visualized on X-ray film (GE Healthcare, Freiburg, Germany) using HRP-conjugated secondary antibodies (Dako; Hamburg, Germany) and the SuperSignal detection system (Thermo Scientific). For quantification, the films were scanned, the grey value of each band was analysed by ImageJ (the National Institutes of Health, Bethesda, MD, USA) and normalized to the grey value of β3-Tubulin.

### Immunohistochemistry

Mouse brains were snap-frozen after removal without perfusion. Tissue was sectioned at 7 μm using a cryostat at −20 °C and a knife temperature of −14 °C. Sections were air-dried on superfrost glass and stored at −80 °C. For staining, sections were defrosted at room temperature (RT) for 60 min and subsequently washed with −20 °C MeOH for 3 min followed by 3 × 10 min of PBS wash. To permeabilize membranes, sections were incubated for 60 min in 0.5% Triton X-100 in PBS at RT and washed 3 × in PBS for 10 min. Following a 60 min incubation in 5% BSA (in PBS) at RT and subsequent PBS washing, sections were incubated in SA5193 antibody (1:1,000, dissolved in 2% bovine-serum albumin, see ref. 36) O/N at 4 °C. The sections were then washed for 3 × 10 min in PBS followed by 120 min of fluorescent antibody staining (1:200, Donkey anti goat-Cy3) in 2% BSA at RT. After the fluorescent antibody staining a wash of 3 × 10 min of PBS was followed by 2 × 10 min wash with PB. The sections were then put for 10 min in DAPI (200 μl in 50 ml 0.1 M PB). This was concluded by a 2 × 10 min PB wash. For Zebrin (Adolase C), we used a different approach after the defrosting of sections at RT. These slices were washed with 10 min 4% PFA followed by 20 min of methanol and subsequently by 2 min PBS and 30 min 100 ml PBS (with 2 ml 30% H_2_O_2_+0.8 ml sodium azide). Here after a wash of 2 min PBS and 2 × 2 min in PBS (with 1 l PBS, 5 g protifar and 1.5 g glycine) sections were incubated in the primary antibody for Adolase C (1:1,000) O/N at 4 °C. The sections were then washed for 3 × 10 min in PBS followed by 90 min of fluorescent antibody staining (1:200, donkey anti goat-Cy3; the Jackson Laboratory, Sacramento, USA, Cat. No 705–165–147) in 2% BSA at RT. After the fluorescent antibody staining a wash of 3 × 10 min of PBS was followed by 2 × 10 min wash with PB. The sections were then put for 10 min in DAPI (ThermoFisher Scientific, Waltham, USA, Cat. No. D3571; 200 μl in 50 ml 0.1 M PB). This was concluded by a 2 × 10 min PB wash. Following PBS washing, the sections were thionin-stained and permount-covered using standard protocols. Images were taken using an upright confocal microscope (LSM 700, Zeiss, Oberkochen, Germany) using 405 nm and 555 nm wavelengths. All immunohistological stainings have successfully been replicated on multiple occasions.

### *Ex vivo* electrophysiology

Following decapitation of mice under isoflurane anaesthesia, the cerebellum was removed into an ice-cold ‘slicing medium', containing (in mM) 240 sucrose, 2.5 KCL, 1.25 Na_2_HPO_4_, 2 MgSO_4_, 1 CaCl_2_, 26 NaHCO_3_ and 10 D-Glucose that was carbogenated continuously (95% O_2_ and 5% CO_2_). Sagittal slices, 250 μm thick, of the cerebellar vermis were cut using a vibrotome (VT1200S, Leica) and put in carbogenated artificial cerebrospinal fluid (ACSF) containing (in mM): 124 NaCl, 5 KCL, 1.25 Na_2_HPO_4_, 2 MgSO_4_, 2 CaCl_2_, 26 NaHCO_3_ and 20 D-Glucose, for at least 1 h at 34±1 °C before the start of the experiment. Slice physiology was done at RT 21±1 °C or 33±1 °C as indicated in the Results section and in the presence of 100 μM picrotoxin except for the sIPSCs recordings. Whole-cell patch clamp recording were performed with an EPC9 amplifier (HEKA Electronics, Lambrecht, Germany). Recordings were excluded if the series (Rs) or input resistances (Ri) changed by >15% during the experiment, which was determined using a hyperpolarizing voltage step relative to the −65 mV holding potential. Data analysis (rise times (10–90% for EPSC and APs)), decay time (tau) for EPSC and IPSC amplitudes, AP threshold (identified by steepest slope in membrane potential prior to AP) and AHP amplitude (minimal membrane potential relative to the AP threshold) were performed using Clampfit software (Molecular Devices).

For whole-cell recordings PCs were visualized using an upright microscope (Axioskop 2 FS, Carl Zeiss) equipped with a × 40 objective. Recording electrodes (3–5 MΩ, 1.65 mm outside diameter (OD) and 1.11 mm interior diameter (ID), World Precision Instruments, Sarasota, FL, USA) were filled with an intracellular solution containing (mM): 120 K-Gluconate, 9 KCL, 10 KOH, 4 NaCL, 10 HEPES, 28.5 Sucrose, 4 Na_2_ATP, 0.4 Na_3_GTP (pH 7.25–7.35 with an osmolarity of 295±5). Note that we adjusted the osmolarity using sucrose^46^,^60^. For the recording of sIPSCs, we used an intracellular solution containing (mM): 150 CsCl, 1.5 MgCl_2_, 0.5 EGTA, 4 Na_2_ATP, 0.4 Na_3_GTP, 10 HEPES, 5 QX314 (pH 7.25–7.35 with an osmolarity of 295±5). For extracellular stimulation of PFs, similar patch electrodes were filled with ACSF and positioned in the upper third of the molecular layer lateral to the patched PC. The stimulation intensity was set to evoke an EPSC of 300±50 pA (typically 3–6 μA stimulation intensity). For PF–PC transmission, we used various inter-stimulus intervals (50–200 ms) (see Fig. 2). For recordings of spontaneously occurring IPSCs (sIPSCs), we used the previously mentioned K^+^-based internal and recorded their occurrence during at least 120 s.

For CF stimulation, similar electrodes (filled with ACSF) were positioned near the patched PC soma in the surrounding granule layer. We selected those recordings in which CF stimuli elicited clear all-or-none responses and lacked the co-activation of PC axons (identifiable by backpropagating APs) for further analysis. For CF elimination, experimental tissue was prepared in a similar way for all age groups. We systematically scanned the granule cell layer to elicit CF responses and recorded PC responses (using an intracellular solution containing (in mM): 115 CsMeO_3_, 20 CsCl, 2.5 MgCl_2_ 10 HEPES, 0.6 EGTA, 4 Na_2_ATP, 0.4 Na_3_GTP, 10 Na-phosphocreatine) at −20 mV holding potential to prevent voltage escape during the CF responses. For CF–PC transmission, we evaluated the paired-pulse ratio at 50 ms stimulus interval. To evaluate the complex spike waveforms, we analysed the amplitude of the Na^+^-spike and the amplitude of the first Ca^2+^-spikelet evoked during the LTD-tetanus^61^. Current clamp recordings were corrected offline for the calculated liquid junction potential (−10.2 mV).

The synaptic (LTP, LTD) and intrinsic plasticity protocols were recorded from lobules 5/6 and conducted as described previously^32^,^46^. In short, for synaptic plasticity all recordings were done in voltage-clamp, except for the tetanus, which consisted of single-pulsed PF-stimulation at 1 Hz for 5 min (LTP) or single-pulsed PF+single-pulsed CF stimulation (5 ms interval) at 1 Hz for 5 min (LTD). We evaluated the synaptic plasticity by the change in PF-EPSC (baseline at 0.05 Hz) relative to the mean value calculated during the last 5 min pre-tetanus. For intrinsic plasticity, we utilized the PF-LTP tetanus (but without bias currents, that is, *I*=0 pA) and evaluated the impact on the number of APs evoked by 300 pA current injections during 500 ms (presented at 0.05 Hz).

### Extracellular PC recordings

*In vivo* recordings were performed as recently described^33^. An immobilizing pedestal was fixed on the skull and a craniotomy (Ø 3 mm) was performed on the occipital bone. After recovery of 5 days, mice were head-fixed and body restrained for recordings. Single-unit recording was identified by the presence of a short SS pause (>6 ms) after each complex spike. PCs were recorded from vermal lobules I–V and X using single barrel (2.0 mm OD, 1.16 mm ID, Harvard Apparatus, MA, USA) and double barrel (theta septum, 1.5 OD, 1.02 ID; World Precision Instruments) boroscilate glass pipettes. *In vivo* recordings were analysed offline using Spiketrain (Neurasmus BV, Rotterdam, The Netherlands, www.neurasmus.com) and custom scripts in MatLab (Mathworks, Natick, MA, USA). The CV is calculated by dividing the s.d. by the mean of the interspike intervals, whereas CV2 is calculated as 2 × |ISI_n+1_−ISI_n_|/(ISI_n+1_+ISI_n_).

### Compensatory eye movements

Mice between 8 and 10 weeks of age were prepared for head-restrained recordings of compensatory eye movements. These types of recordings have been described in detail previously^32^. To head restrain the mice during the eye movement task, a small pedestal was attached using Optibond primer and adhesive (Kerr, Bioggio, Switzerland) under isoflurane anaesthesia in O_2_ (induction with 4% and maintained at 1.5% concentration). After a recovery period of 2–3 days, mice were head-restrained by fixation using the pedestal in the experimental set-up. A round screen with a random dotted pattern (‘drum') surrounded the mouse during the experiment. The OKR, VOR and the light-guided VOR (VVOR) were induced using a sinusoidal rotation of the drum in light (OKR), rotation of the table in the dark (VOR) or the rotation of the table (VVOR) in the light. The motor behaviour was assessed by rotating the table and/or drum at 0.1–1 Hz with a fixed 5° amplitude. To evaluate motor learning, a mismatch between visual and vestibular input was created. Rotating both the visual and vestibular stimuli in phase (at the same amplitude) induced a decrease of gain; rotating the drum at greater amplitude relative to the table induced the so-called phase reversal of the VOR (day 1, 5° phase difference; day 2, 7.5°; day 3–4, 10°). Rotating the visual and vestibular stimuli out of phase (at the same amplitude) induced the VOR gain increase. All training protocols were induced at 0.6 Hz with table rotation amplitude of 5°. For eye illumination during the experiments, two table-fixed infrared emitters (output 600 mW, dispersion angle 7°, peak wavelength 880 nm) and a third emitter, which produced the tracked corneal reflection, were mounted to the camera and aligned horizontally with the optical axis of the camera. Eye movements were recorded with eye-tracking software (ETL-200, ISCAN systems, Burlington, NA, USA). Gain and phase values of eye movements were calculated using Matlab.

### Eye-blink conditioning

Mice of 12–15 weeks of age were prepared for head-restrained eye-blink conditioning^39^. In short, a small brass pedestal was attached to the skull using Optibond primer and adhesive and Charisma (Heraeus Kulzer, Armonk, NY, USA), under isoflurane anaesthesia. Three to five days after surgery, mice were habituated during two short (30–45 min) sessions on 2 days in a sound- and light-isolating chamber which houses the eye-blink set-up. During these sessions, mice were head-fixed and suspended over a foam cylindrical treadmill. While no stimuli were presented, a 27.5 gauge needle through which unconditional stimulus (US) air-puffs are delivered was positioned at 5 mm from the centre of the left cornea, a green LED (ø 5 mm) that delivers the CS was placed 5 cm in front of the mouse and an GMR magnetometer (NVE, Eden Prairie, MN, USA) was fixed above the left eye. During 5 subsequent acquisition training days, this sensor measured the distance of a miniscule magnet (1.5 × 0.7 × 0.5 mm) that was placed on the left lower eyelid with high accuracy, while 200 paired trials were presented, usually spaced 10±2 s apart, plus the time needed for a sporadic unstable eyelid to stabilize in open position. Each paired trial consisted of a 280 ms green LED CS, co-terminating with a 30 ms air puff (30 psi, through an MPPI-3 pressure injector; ASI, Eugene, OR, USA). All experiments were performed at approximately the same time of day by the same experimenter. Individual eye-blink traces were analysed using custom LabVIEW (National Instruments, Austin, TX, USA) or Matlab scripts. Trials with significant activity in a 500 ms pre-CS baseline period were regarded as invalid for further analysis. Valid trials were aligned by making the mean of their baseline activity zero, and the average amplitude of all post-US unconditioned blink responses was used to denote 100% eyelid closure. From this, the average eyelid closure as a percentage from baseline to full closure at the end of the CS–US interval was calculated over all valid trials. To calculate percentage of CRs, trials were judged to contain a CR if the eye closed for >5% between 50 and 250 ms after CS and the CR reached its peak after 100 ms.

### ErasmusLadder

The ErasmusLadder (Noldus, Wageningen, Netherlands) is a fully automated system consisting of a horizontal ladder between two shelter boxes. It has 37 rungs on each side, spaced 15 mm apart, and attached to custom-made pressure sensors that are continuously monitored. To create a left-right alternating pattern, even rungs on one side and odd rungs on the other side are elevated by 6 mm. Prototype testing revealed that optimum forepaw displacement for mice is about 6 cm in a single step at medium high velocity, which is the distance between three consecutive elevated rungs and is defined as an efficient step in the text (for more details, see ref. 37). It is clear from previous studies that mice improve their walking efficiency over training sessions by increasing the number of efficient steps relative to steps of lower sizes^37^. In the current study, mice (male, 15–18 weeks old) were tested in six daily sessions consisting of two unperturbed sessions, one session with a fixed obstacle in the middle of the ladder and three perturbed sessions. During the first three sessions, mice were trained to walk between 2 shelter boxes for 50 trials each day. In the perturbed sessions, a sudden appearance of a rising rung on the right side of the mouse was used as the US. A 15 kHz tone was used as the CS and preceded the US by 200 ms in CS–US paired trials. CS-only trials and paired trials were randomly distributed among 50 trials. There were twice as many paired trials as CS-only trials. Step length and step time were defined as the distance and time between two consecutive touches from the right front limb. To estimate motor adaptation in CS-only trials, we calculated step speed (step length/step time) using only the steps within 1 s before and 1 s after the CS. The speed ratio during conditioning was defined as the speed post-CS divided by the speed pre-CS.

### General behavioural analyses

Behavioural experiments were performed using *L7-Shank2*^−/−^ and WT littermate controls aged 8–16 weeks during the light period of their diurnal cycle. The mice used in the general behavioural experiments described here underwent multiple tests.

For the PhenoTyper test, mice were placed in a homecage-like apparatus (Noldus) with ad libitum access to food and water, and left to explore for 30 min. Locomotion was recorded using automated software (Noldus Ethovision XT 11) and distance and speed were calculated.

For the open field test, mice were placed in a novel circular, brightly-lit 110-cm-diameter open arena for 10 min. Locomotion was recorded using automated software (Noldus Ethovision XT 11) and total distance travelled, as well as average speed were calculated. During analysis the arena was subdivided into three concentric zones named the inner (25 cm), middle (15 cm) and outer zone (15 cm), and percentage of time in each zone was calculated.

For the three-chamber social interaction test, age- and gender-matched WT target subjects (Stranger 1 and 2) were habituated for 5 consecutive days before beginning of testing by being placed inside round metal-wired cages. On the test day, experimental mice were placed in the central chamber of a clear Plexiglas box (60 × 35 cm) divided into three inter-connected chambers. After habituation for 5 min, an unfamiliar mouse (Stranger 1; S1) was introduced into a wire cage in one of the side-chambers and an empty wire cage in the other side-chamber. The dividers were then raised and the test mouse was allowed to freely explore all three chambers over a 5-min session. Next, the mouse remained in the chamber with stranger 1 for an additional 5-min session. Subsequently, a novel stranger mouse (Stranger 2 ) was placed in the previously empty wire cage and again the test mouse was left to explore for 5 min. Time spent in each chamber, as well as overall locomotion, was calculated using automated software (Noldus Ethovision XT 11). Preference indices were calculated by subtracting the time spent with the empty wire cage from the time spent with stranger 1 (S1−E), and subtracting the time spent with stranger 1 from time spent with stranger 2 (S2–S1).

For the T-maze spontaneous alternation test, mice were placed at the base of a T-maze (arm length 50 cm) and were given the choice to freely explore either the right or left arm of the maze for 10 consecutive trials. A choice was assumed to be made when mice stepped into an arm with all four paws. At that moment, the gate to that arm was closed and the animal was allowed to explore the arm for 5 s. Then, the mouse was gently placed back at the base of the T-maze for the next trial. When the mouse chose a similar arm at two consecutive trials, this was scored as number of repeats, indicative of repetitive behaviour.

For the grooming test, mice were removed from their homecage, received a single puff of water spray and placed in clean transparent cages (15 × 15 × 20 cm) under bright light for 15 min. Behaviour was recorded with a high-speed camera (30 Hz full frame rate), and time spent grooming was scored by two independent raters. All types of grooming—paw licking, nose and head self-grooming, body grooming, leg grooming and tail/genital grooming—were scored.

For the marble-burying test, Makrolon cages (50 × 26 × 18 cm) were filled with 4 cm of bedding material and 20 glass marbles, which were arranged in an equidistant 4 × 5 grid. Animals were given access to the marbles for 30 min. Marbles 100% covered by bedding were scored as buried and marbles covered partially contributed 50% to the total score.

For the olfactory test, during the habituation phase a piece (1.25 g) of cookie was put into the subject's cage each day for 3 consecutive days, and checked for complete consumption the following day. For 24 h before the test phase, mice were completely food-deprived. Subjects were placed in a clean Makrolon cage (50 × 26 × 18 cm) with 4 cm of clean bedding, and allowed to habituate for 5 min. Then, a piece of cookie (1.25 g) was hidden in a random corner in the cage at 1 cm depth. Latency to find the cookie was recorded for a maximum of 15 mins (900 s).

### Data analysis

In the text, mean±s.d. values are presented, in the figures s.e.m. values are reported, and *P* values <0.05 are considered significantly different. Two-sided Student's *t*-tests were performed, unless stated otherwise. For a complete representation of the data, we have included a detailed overview of all statistics in Supplementary Table 1. In addition, since the L7-Cre line was shown to reveal germ-line deletions (see also ref. 44) and since the potential impact of cerebellar PCs on general cognitive tests is widely debated^62^,^63^, we have checked for heterozygous germ-line deletions in our L7-Shank2 mice and statistically excluded the possibility that they could have influenced our conclusions on the non-cerebellar paradigms (Supplementary Table 1).

### Data availability

In addition to our Supplementary Data File, which contains data points that support the findings of this study, data sets are available from the corresponding authors upon request.

## Additional information

**How to cite this article:** Peter, S. *et al.* Dysfunctional cerebellar Purkinje cells contribute to autism-like behaviour in *Shank2*-deficient mice. *Nat. Commun.* 7:12627 doi: 10.1038/ncomms12627 (2016).

## Supplementary Material

Supplementary Information Supplementary Figures 1-7 and Supplementary Table 1
