# Supplementary material for: Dysfunctional cerebellar Purkinje cells contribute to autism-like behaviour in Shank2-deficient mice
Source: Nat Commun. 2016 Sep 1;7:12627. doi: 10.1038/ncomms12627 (PMC5025785; doi:10.1038/ncomms12627)
Supplement: Supplementary Information — Supplementary Figures 1-7 and Supplementary Table 1 [file ncomms12627-s1.pdf]

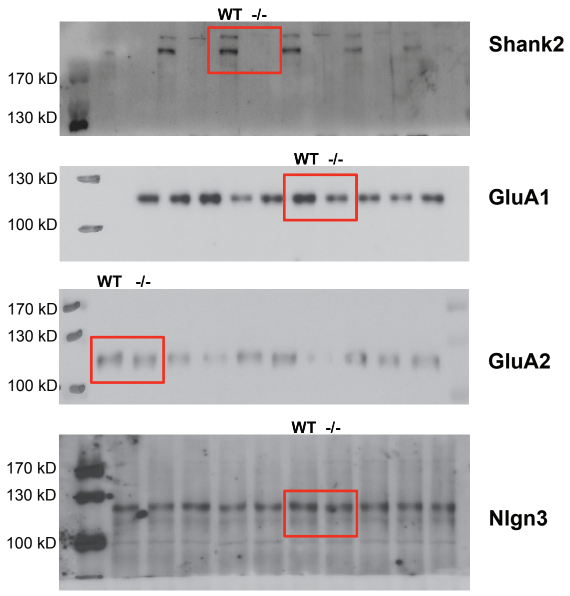

Supplementary Figure 1 Full western blots with size markers related to Figure 1d.

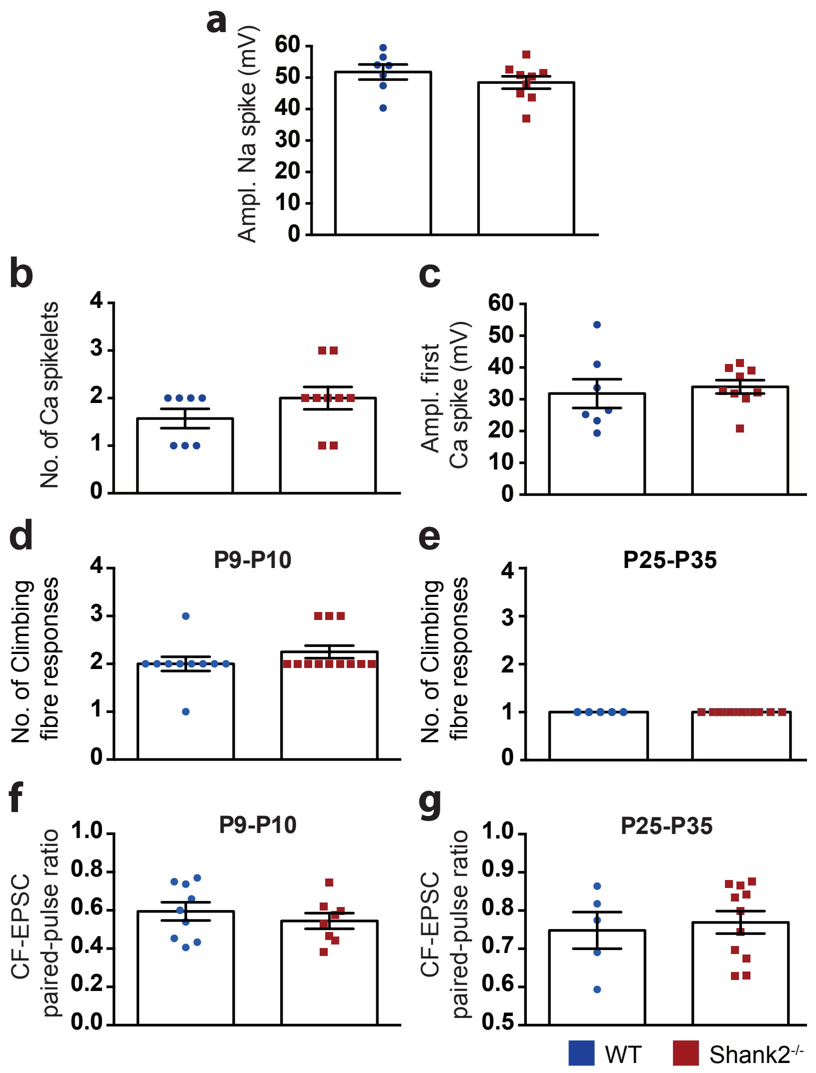

Supplementary Figure 2 Ex vivo climbing fibre activity shows no significant differences between Shank2$^{-/-}$ and WT mice. (**a-c**) No difference in Na spike amplitude (P=0.3) (**a**), nor in number of Ca spikelets (P=0.2) (**b**) or amplitude of the first Ca spike (P=0.7) (**c**), between WT (n=7/6) and Shank2$^{-/-}$ (n=9/4). (**d,e**) Number of climbing fibre responses was similar between Shank2$^{-/-}$ and WT mice at P9-10 (WT, n=10/3; Shank2$^{-/-}$, n=12/3, P=0.2)) and at P25-35 (WT, n=5/3; Shank2$^{-/-}$, n=12/3, P=1.0). (**f,g**) Complex spike paired pulse depression was similar between Shank2$^{-/-}$ and WT mice at P9-10 (WT, n=9/3; Shank2$^{-/-}$, n=8/3, P=0.5) and at P25-35 (WT, n=5/3; Shank2$^{-/-}$, n=11/3, P=0.6).Statistical test used was a two-sided t-test unless stated otherwise.

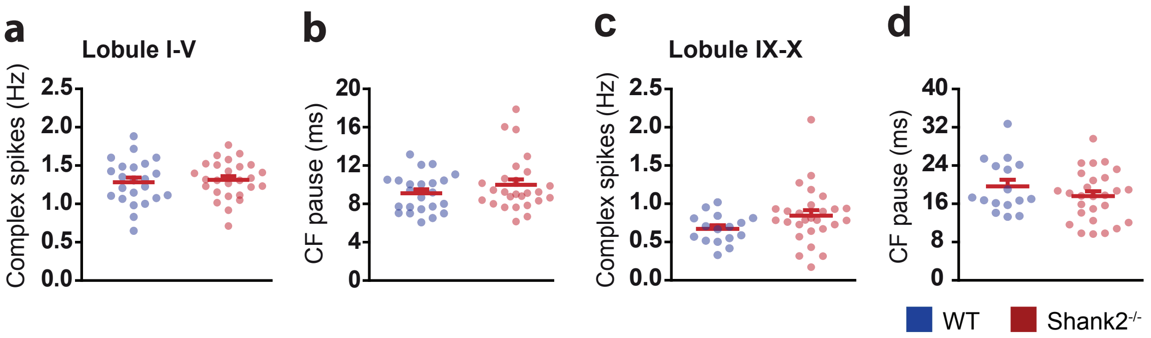

Supplementary Figure 3 In vivo complex spike activity shows no significant differences between Shank2$^{-/-}$ and WT mice. (**a,b**) Complex spike firing frequency (P=0.7) and CF pause (P=0.2) were not different between Shank2$^{-/-}$ and WT mice in anterior lobules I-V (WT, n=23/3, cells/animals; Shank2$^{-/-}$, n=26/3). (**c,d**) Similarly, complex spike firing rate (P=0.1) and CF pause (P=0.2) did not differ between mutant (n=27/3) and WT (n=16/3) in posterior lobules IX-X. Error bars denote SEM. Statistical test used was a two-sided t-test unless stated otherwise.

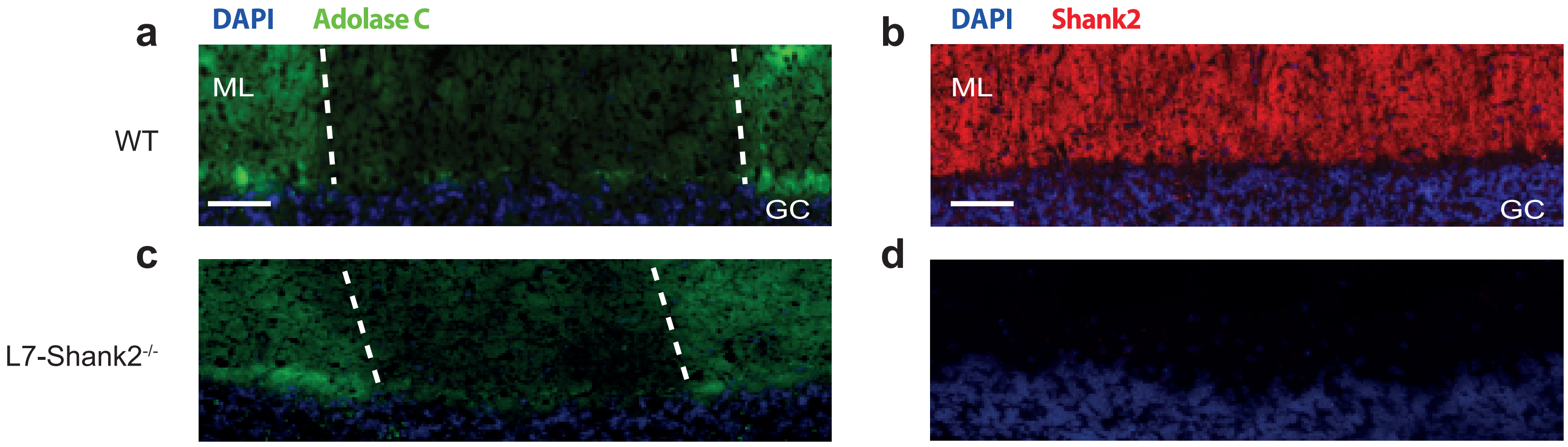

Supplementary Figure 4 Cerebellar Zebrin and Shank2 immunohistology. (**a,c**) Coronal section of Crus 1 showing distinctive Zebrin patterning in both WT and L7-Shank2-/-. (**b,d**) Coronal section of Crus 1 showing a uniform presence of Shank2 in WT, but a complete absence of Shank2 in L7-Shank2-/-. Scale bar: 50 μm.

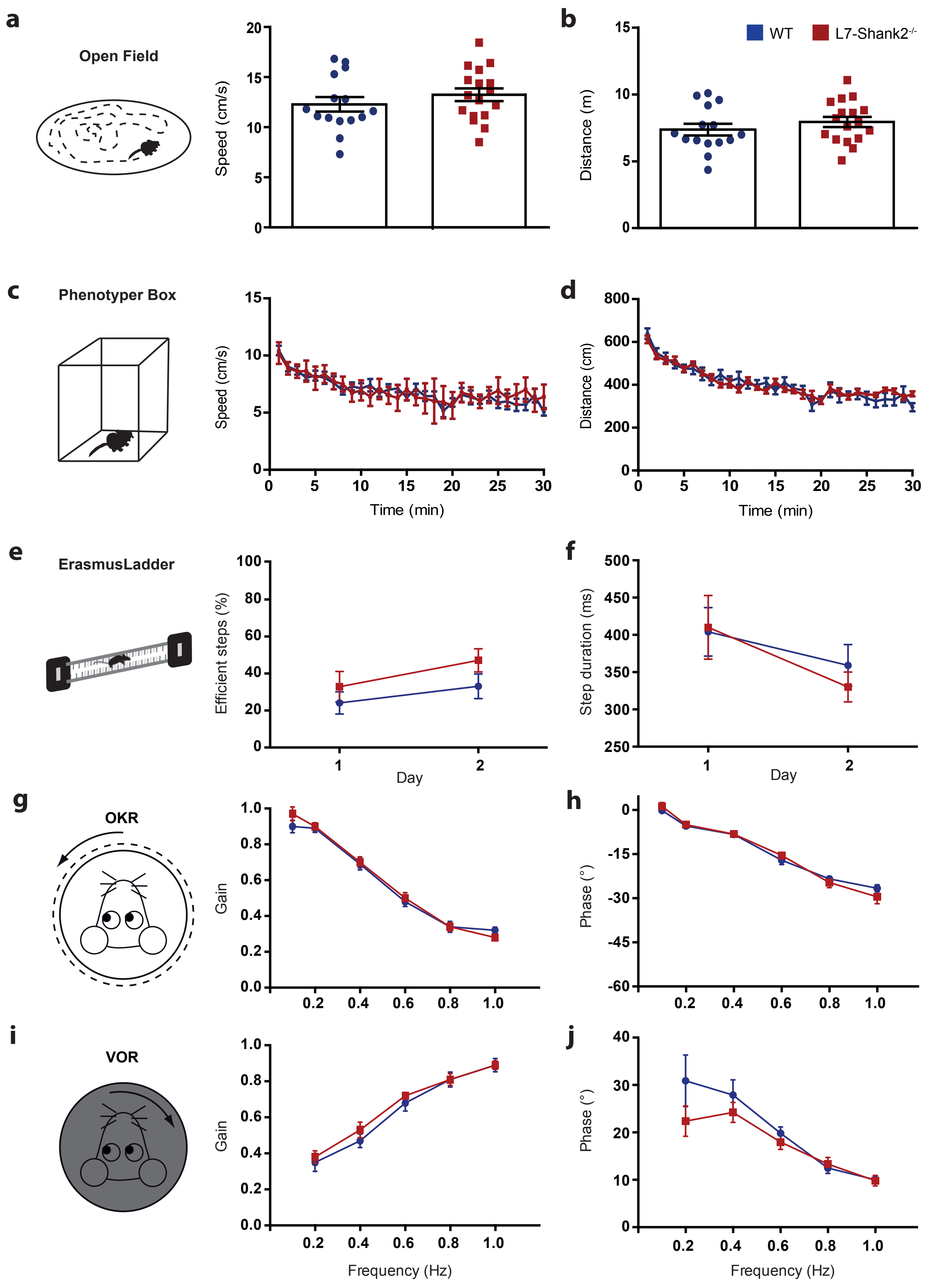

Supplementary Figure 5 L7-Shank2$^{-/-}$ mice have normal basic motor performance. (**a**) In the open field experiment mice were allowed to move around freely. The speed of movement (P=0.3, t-test) and (**b**) distance travelled (P=0.3, t-test) during 10 min was not different (WT, n=15; L7-Shank2$^{-/-}$, n=17). (**c**,**d**) These results were confirmed in the phenotyper box for both speed (P=0.5) and distance (P=0.5) (WT, n=16; L7-Shank2$^{-/-}$, n=16). (**e**) ErasmusLadder revealed no difference in the percentage of efficient steps (P=0.3) or (**f**) the duration of these steps (P=0.8) over the course of two days (WT, n=10; L7-Shank2$^{-/-}$, n=6). (**g**,**h**) Optokinetic reflex (OKR) using different frequencies of drum movement revealed no differences in gain (P=0.6) or phase (P=0.9) (WT, n=9; L7-Shank2$^{-/-}$, n=9). (**i**,**j**) Similarly, the vestibulo-ocular reflex (VOR) was not affected in either gain (P=0.4) or phase (P=0.2) (WT, n=8; L7-Shank2$^{-/-}$, n=9). Data are represented as mean ± SEM. Statistical test used was a repeated-measures ANOVA unless stated otherwise.

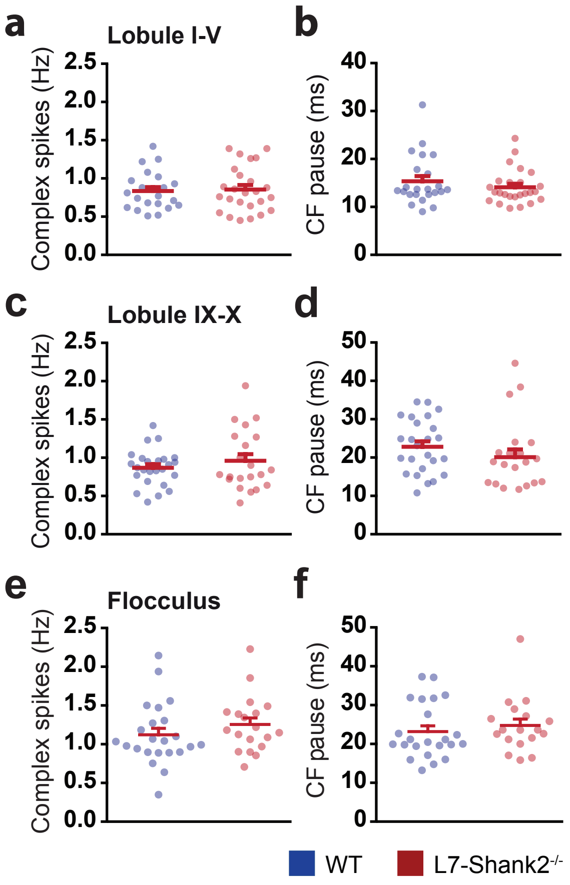

Supplementary Figure 6 In vivo complex spike firing characteristics in L7-Shank2$^{-/-}$ Purkinje cells. (**a,b**) No significant differences in complex spike firing frequency (P=0.6) or climbing fibre pause (P=0.3) in anterior lobules (I-V) between WTs (n = 23/3, cells/animals) and L7-Shank2$^{-/-}$ mice (n = 25/3). (**c,d**) Similarly, in posterior lobules IX-X, complex spike firing frequency (P=0.3) and climbing fibre pause (P=0.3) was not different between WTs (n=25/3) and mutants (n=21/3). (**e,f**) Also located in the posterior cerebellum, Purkinje cells from flocculus showed neither firing rate (P=0.3) nor climbing fibre pause (P=0.5) differences between L7-Shank2$^{-/-}$ (n=19/2) and WTs (n=23/2). Error bars denote SEM. Statistical test used was a two-sided t-test unless stated otherwise.

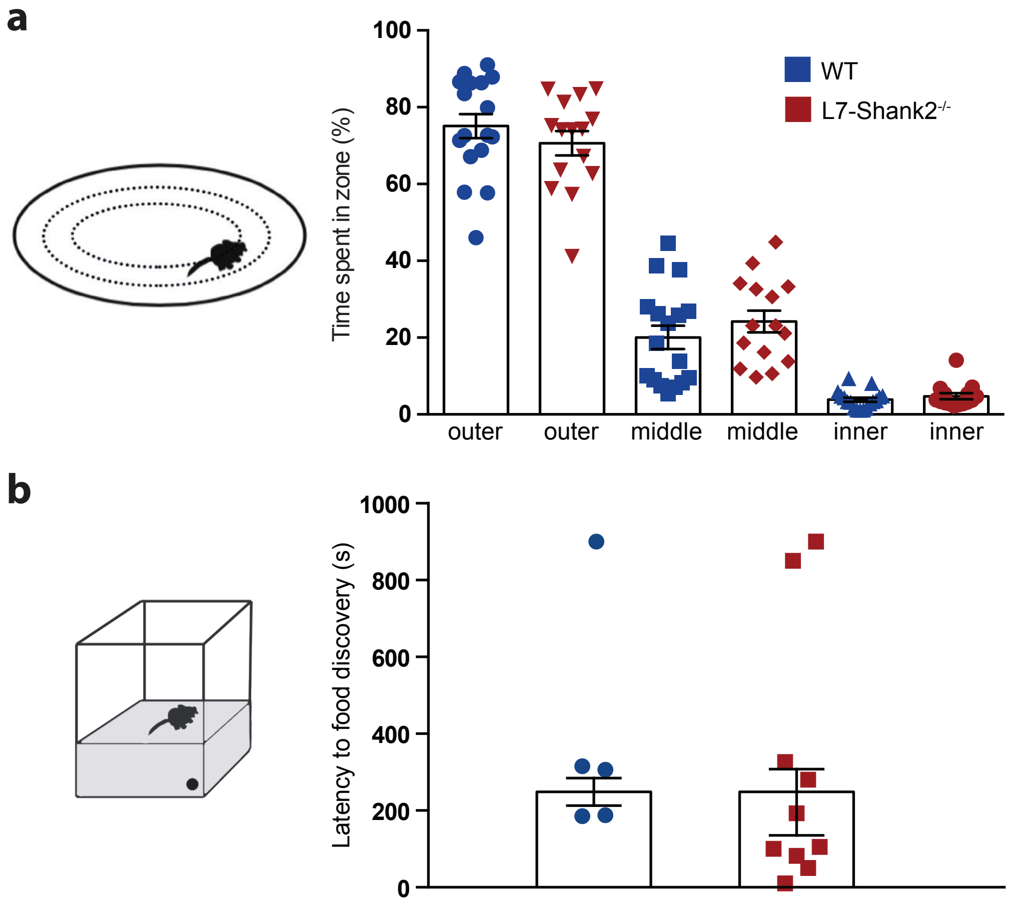

Supplementary Figure 7 (**a**) In the open field test, WT (n=17) and L7-Shank2$^{-/-}$ mice (n=15) showed no differences (P=0.7, Chi-square) in time spent in the inner, middle, or outer zones. (**b**) In the olfaction test, WT (n=5) and L7-Shank2$^{-/-}$ mice (n=10) showed no significant differences (P=0.6, t-test) in time taken to find buried food.Error bars denote SEM.

Supplementary Table 1

**Mann-Whitney U-tests**									
**Figure**	**Parameter**	**N**	**Mean**	**SEM**	**SD**	**Cohen's *d***	**U**	**Sum of ranks**	**P-Value**
Fig. 1a	Spine density/µm WT	97 dendrites	1.93	0.08	0.74			9.499	
	Spine density/µm KO	89 dendrites	1.82	0.07	0.67	0.17	3.887	7.892	0.2420
Fig. 1a	Spine length WT	748 spines	1.34	0.03	0.77			512.348	
	Spine length KO	639 spines	1.32	0.02	0.50	0.02	232.222	450.230	0.3630
Fig. 1a	Spine width WT	748 spines	0.72	0.02	0.45			518.914	
	Spine width KO	639 spines	0.71	0.01	0.32	0.04	238.788	443.665	0.9790
Fig. 1c	PSD length WT	226 PSDs	313.30	6.46	97.11			54.610	
	PSD length KO	243 PSDs	305.30	6.17	96.24	0.08	25.960	55.610	0.3080
Fig. 1c	PSD thickness WT	223 PSDs	26.19	0.40	5.95			50.838	
	PSD thickness KO	233 PSDs	25.99	0.35	5.34	0.04	25.860	53.358	0.9340

**Two-sided t-tests**									
**Figure**	**Parameter**	**N**	**Mean**	**SEM**	**SD**	**Cohen's *d***	**t**	**df**	**P-Value**
Fig. 1d	GluA1 intensity WT	12 synaptosomes	1.00	0.11	0.37				
	GluA1 intensity KO	6 synaptosomes	0.63	0.09	0.23	1.24	2.23	16	0.0410
Fig. 1d	GluA2 intensity WT	11 synaptosomes	1.00	0.10	0.32				
	GluA2 intensity KO	5 synaptosomes	0.58	0.05	0.11	1.96	2.96	14	0.0140
Fig. 1d	Nlgn3 intensity WT	12 synaptosomes	1.00	0.11	0.37				
	Nlgn3 intensity KO	6 synaptosomes	0.84	0.13	0.33	0.47	0.91	16	0.3770

Two-sided t-tests

Figure	Parameter	N	Mean	SEM	SD	Cohen's *d*	t	df	P-Value
Fig. 2b	Holding current pA (WT)	9 cells, 6 mice	-389.40	34.02	102.00				
	Holding current pA (KO)	7 cells, 6 mice	-388.40	42.98	113.70	-0.01	0.02	14	0.9851
Fig. 2c	Input Resistance MΩ (WT)	9 cells, 6 mice	67.23	5.61	16.81				
	Input Resistance MΩ (KO)	7 cells, 6 mice	69.08	4.69	12.40	-0.13	0.24	14	0.8112
Fig. 2d	Rise time ms (WT)	9 cells, 6 mice	2.12	0.24	0.71				
	Rise time ms (KO)	7 cells, 6 mice	1.71	0.21	0.55	0.65	1.25	14	0.2318
Fig. 2e	Decay time ms (WT)	9 cells, 6 mice	9.66	0.27	0.80				
	Decay time ms (KO)	7 cells, 6 mice	9.30	0.12	0.31	0.64	1.11	14	0.2853
Fig. 2j	AP threshold mV (WT)	10 cells, 6 mice	-51.40	1.23	3.89				
	AP threshold mV (KO)	11 cells, 6 mice	-51.00	1.05	3.49	-0.11	0.25	19	0.8066
Fig. 2k	AP amplitude mV (WT)	10 cells, 6 mice	39.78	1.82	5.77				
	AP amplitude mV (KO)	11 cells, 6 mice	35.92	1.75	5.80	0.67	1.53	19	0.1433
Fig. 2l	AP Half-Width ms (WT)	10 cells, 6 mice	0.29	0.01	0.02				
	AP Half-Width ms (KO)	11 cells, 6 mice	0.30	0.01	0.03	-0.16	0.36	19	0.7207
Fig. 2m	AHP amplitude mV (WT)	10 cells, 6 mice	6.48	0.52	1.63				
	AHP amplitude mV (KO)	11 cells, 6 mice	7.55	0.50	1.66	-0.65	1.49	19	0.1541
Fig. 2o	Slope Hz/100pA (WT)	10 cells, 5 mice	16.20	0.70	2.20				
	Slope Hz/100pA (KO)	11 cells, 5 mice	16.18	0.69	2.17	0.01	0.02	19	0.9854

Repeated Measures ANOVAs

Figure	Outcome Variable	N	Parameter	Type III sum of squares	df	mean square	F	P-Value	Partial η2
Fig. 2g	EPSC amplitude	WT: 11 cells/3 mice; KO: 15/3							
			Intercept	7.715.423	1	7.715.423	111.68		
			Genotype	1.526	1	1.526	0.02	0.8830	0.00
Fig. 2h	Paired Pulse facilitation	WT: 11 cells/3 mice; KO: 15/3							
			Intercept	299	1	299	3070.37		
			Genotype	0	1	0	1.44	0.2430	0.06
			Error	2	23	0			
Fig. 2o	Hz	WT: 10 cells/5 mice; KO: 11/5							
			Intercept	826.245	1	826.245	343.49		
			Genotype	5.875	1	5.875	2.44	0.1350	0.11
			Error	45.703	19	2.405			

Two-sided t-tests									
**Figure**	**Parameter**	**N**	**Mean**	**SEM**	**SD**	**Cohen's *d***	**t**	**df**	**P-Value**
Fig. 3c	sIPSCs Hz (WT) - Anterior	25 cells, 3 mice	8.31	1.18	5.92				
	sIPSCs Hz (KO) - Anterior	20 cells, 3 mice	12.16	1.21	5.39	-0.68	2.25	43	0.0295
Fig. 3d	Amplitude pA (WT) - Anterior	25 cells, 3 mice	53.16	4.88	24.42				
	Amplitude pA (KO) - Anterior	20 cells, 3 mice	65.16	6.60	29.53	-0.44	1.49	43	0.1426
Fig. 3e	sIPSCs Hz (WT) - Posterior	19 cells, 3 mice	14.24	1.60	6.98				
	sIPSCs Hz (KO) - Posterior	19 cells, 3 mice	21.48	2.02	8.79	-0.92	2.81	36	0.0079
Fig. 3f	Amplitude pA (WT) - Posterior	19 cells, 3 mice	58.94	4.36	19.00				
	Amplitude pA (KO) - Posterior	19 cells, 3 mice	63.99	5.67	24.73	-0.23	0.71	36	0.4854
Fig. 3c,e	sIPSCs Hz (KO) - Anterior	20 cells, 3 mice	12.16	1.21	5.39				
	sIPSCs Hz (KO) - Posterior	19 cells, 3 mice	21.48	2.02	8.79	-1.31	4.10	34	0.0002
Fig. 3i	Simple Spikes Hz (WT) - Anterior	23 cells, 3 mice	88.18	3.90	18.69				
	SS Hz (KO) - Anterior	26 cells, 3 mice	76.26	2.31	11.80	0.78	2.70	47	0.0096
Fig. 3j	SS CV (WT) - Anterior	23 cells, 3 mice	0.48	0.01	0.06				
	SS CV (KO) - Anterior	26 cells, 3 mice	0.49	0.02	0.12	-0.11	0.35	47	0.7274
Fig. 3k	SS CV2 (WT) - Anterior	23 cells, 3 mice	0.45	0.01	0.04				
	SS CV2 (KO) - Anterior	26 cells, 3 mice	0.48	0.02	0.08	-0.47	1.52	47	0.1348
Fig. 3l	SS Hz (WT) - Posterior	16 cells, 3 mice	52.61	3.18	12.71				
	SS Hz (KO) - Posterior	27 cells, 3 mice	50.26	2.47	12.86	0.18	0.58	41	0.563
Fig. 3m	SS CV (WT) - Posterior	16 cells, 3 mice	0.30	0.02	0.08				
	SS CV (KO) - Posterior	27 cells, 3 mice	0.38	0.02	0.09	-0.89	2.76	41	0.0086
Fig. 3n	SS CV2 (WT) - Posterior	16 cells, 3 mice	0.29	0.02	0.07				
	SS CV2 (KO) - Posterior	27 cells, 3 mice	0.40	0.02	0.11	-1.35	3.94	41	0.0003

Repeated Measures ANOVAs									
Figure	Outcome Variable	N	Parameter	Type III sum of squares	df	mean square	F	P-Value	Partial η2
Fig. 4b	EPSC LTP amplitude (WT)	7 cells, 6 mice							
			Intercept	1.680.008	1	1.680.008	1494.48		
			Pre/Post Induction	15.866	1	15.866	14.11	0.0027	0.54
			Error	13.490	12	1.124			
Fig. 4b	EPSC LTP amplitude (KO)	12 cells, 6 mice							
			Intercept	1.716.242	1	1.716.242	2205.34		
			Pre/Post Induction	1.108	1	1.108	1.42	0.2502	0.08
			Error	12.452	16	778			
Fig. 4b	EPSC LTP amplitude	WT: 7 cells/6 mice; KO: 12/6							
			Intercept	4.879.383	1	4.879.383	1347.11		
			Genotype	36.801	1	36.801	10.16	0.0066	0.42
			Error	50.709	14	3.622			
Fig. 4d	EPSC LTD amplitude (WT)	9 cells, 6 mice							
			Intercept	1.335.607	1	1.335.607	1475.70		
			Pre/Post Induction	32.034	1	32.034	35.39	0.0000	0.69
			Error	14.481	16	905			
Fig. 4d	EPSC LTD amplitude (KO)	7 cells, 6 mice							
			Intercept	1.131.917	1	1.131.917	1013.69		
			Pre/Post Induction	21.249	1	21.249	19.03	0.0009	0.61
			Error	13.400	12	1.117			
Fig. 4d	EPSC LTD amplitude	WT: 9 cells/6 mice; KO: 7/6							
			Intercept	2.495.061	1	2.495.061	530.17		
			Genotype	7	1	7	0.00	0.9698	0.00
			Error	65.886	14	4.706			
Fig. 4f	Normalized spike output (WT)	5 cells, 4 mice							
			Intercept	1.477.506	1	1.477.506	561.08		
			Pre/Post Induction	37.741	1	37.741	14.33	0.0053	0.64
			Error	21.067	8	2.633			
Fig. 4f	Normalized spike output (KO)	5 cells, 4 mice							
			Intercept	1.030.884	1	1.030.884	210.74		
			Pre/Post Induction	2.142	1	2.142	0.44	0.5268	0.05
			Error	39.134	8	4.892			
Fig. 4f	Normalized spike output	WT: 5 cells/4 mice; KO: 5/4							
			Intercept	3.419.536	1	3.419.536	418.42		
			Genotype	68.396	1	68.396	8.37	0.0201	0.51
			Error	65.381	8	8.173			

Repeated Measures ANOVAs

Figure	Outcome Variable	N	Parameter	Type III sum of squares	df	mean square	F	P-Value	Partial η2
Fig. 6b	Avg. speed ratio (%)	WT: 9 mice; L7-KO: 6							
			Intercept	55.6	1	55.6	313.29		
			Genotype	1.3	1	1.3	7.30	0.0180	0.359
			Error	2.3	13	0.2			
Fig. 6d	Normalized gain (%)	WT: 7 mice; L7-KO: 8							
			Intercept	110.7	1	110.7	646.01		
			Genotype	1.9	1	1.9	10.97	0.0060	0.458
			Error	2.2	13	0.2			
Fig. 6f	Phase reversal (°; Day 2)	WT: 8 mice; L7-KO: 10							
			Intercept	52.966.6	1	52.966.6	318.83		
			Genotype	798.0	1	798.0	4.80	0.0470	0.270
			Error	2.159.7	13	166.1			
Fig. 6f	Phase reversal (Day 3)	WT: 8 mice; L7-KO: 9							
			Intercept	155.981.8	1	155.981.8	138.18		
			Genotype	18.007.5	1	18.007.5	15.95	0.0013	0.533
			Error	15.803.9	14	1.128.9			
Fig. 6f	Phase reversal (Day 4)	WT: 8 mice; L7-KO: 9							
			Intercept	312.466.3	1	312.466.3	188.16		
			Genotype	62.205.6	1	62.205.6	37.46	0.0000	0.714
			Error	24.909.6	15	1.660.6			
Fig. 6f	Phase reversal (Day 5)	WT: 8 mice; L7-KO: 8							
			Intercept	540.417.4	1	540.417.4	78.27		
			Genotype	165.103.6	1	165.103.6	23.91	0.0003	0.648
			Error	89.756.4	13	6.904.3			
Fig. 6h	Conditioned response %	WT: 11 mice; L7-KO: 10							
			Intercept	154.229.1	1	154.229.1	165.39		
			Genotype	13.256.8	1	13.256.8	14.22	0.0013	0.428
			Error	17.718.4	19	932.5			
Fig. 6i	CR Amplitude (CS-US interval)	WT: 11 mice; L7-KO: 10							
			Intercept	2.5	1	2.5	72.88		
			Genotype	0.5	1	0.5	15.42	0.0009	0.448
			Error	0.6	19	0.0			

Descriptive statistics: Erasmus Ladder Speed ratio

		Day 1			Day 2			Day 3		
Figure	Genotype	Mean	SEM	SD	Mean	SEM	SD	Mean	SEM	SD
Fig. 6b	WT	1.125	0.09	0.28	1.33	0.13	0.40	1.47	0.13	0.38
	L7-KO	0.886	0.12	0.29	1.00	0.08	0.20	1.00	0.07	0.17

Descriptive statistics: VOR Phase reversal

		WT			L7-KO		
Figure	Day	Mean	SEM	SD	Mean	SEM	SD
Fig. 6f	1	16.81	1.09	3.07	17.35	1.34	4.24
		16.00	1.11	3.15	18.67	1.46	4.61
		18.09	1.61	4.56	19.39	1.27	4.02
		14.66	2.28	6.45	20.47	1.80	5.70
		20.35	2.25	6.37	19.22	1.65	5.21
		16.31	1.51	4.28	18.04	2.03	6.41
		19.95	1.88	5.32	19.26	1.84	5.83
	2	18.13	0.70	1.97	17.10	1.33	4.22
		20.04	3.21	9.09	19.08	2.14	6.76
		23.78	1.80	5.10	18.34	2.88	9.12
		28.70	1.71	4.85	19.98	2.00	6.32
		32.22	2.01	5.69	19.81	2.89	9.13
		26.12	1.75	4.96	21.40	3.07	9.70
		32.49	3.09	8.75	21.52	2.68	8.48
	3	28.47	1.99	5.63	18.81	1.87	5.92
		46.96	8.08	22.86	23.16	2.08	6.57
		43.47	7.82	22.13	27.09	3.39	10.72
		52.21	6.42	18.15	24.66	2.00	6.32
		55.68	7.98	22.57	24.80	3.55	11.22
		63.05	11.67	33.01	23.54	3.54	11.20
		60.16	9.68	27.37	23.97	2.10	6.65
	4	45.79	5.18	14.65	20.61	1.50	4.73
		62.86	9.88	27.93	26.39	2.95	9.33
		86.09	12.72	35.99	30.32	2.58	8.17
		78.48	11.60	32.81	26.82	3.74	11.83
		80.80	9.42	26.65	32.39	4.00	12.64
		83.90	13.69	38.73	33.47	2.82	8.93
		81.72	11.36	32.13	29.82	3.18	10.06
	5	76.87	16.97	48.00	18.20	2.86	8.10
		115.98	16.76	47.39	29.05	3.55	10.04
		119.39	14.91	42.16	30.97	3.73	10.55
		112.58	15.56	44.01	33.73	4.13	11.69
		119.35	18.32	51.83	42.98	6.75	19.10
		123.48	16.44	46.51	38.53	5.56	15.72
		112.04	14.36	40.62	31.66	5.94	16.79

Descriptive statistics: VOR Gain increase

Figure	Genotype	Minute	Mean	SEM	SD
Fig. 6d	WT	0	1.000	0.074	0.195
		10	1.184	0.121	0.320
		20	1.304	0.115	0.305
		30	1.342	0.073	0.194
		40	1.404	0.103	0.272
		50	1.228	0.151	0.399
	L7-KO	0	1.000	0.032	0.089
		10	0.994	0.029	0.082
		20	1.056	0.063	0.179
		30	1.068	0.069	0.194
		40	1.111	0.065	0.184
		50	1.022	0.059	0.167

Descriptive statistics: Eyeblink conditioning

		WT			L7-KO		
Figure	Day	Mean	SEM	SD	Mean	SEM	SD
Fig. 6h	1	4.50	1.37	4.53	3.85	1.41	4.66
	2	33.05	8.49	28.17	6.90	2.03	6.75
	3	57.14	9.17	30.40	24.30	4.75	15.74
	4	73.14	6.75	22.40	39.20	5.96	19.76
	5	80.27	4.52	14.98	61.35	8.37	27.77
Fig. 6i	1	0.01	0.01	0.00	0.01	0.01	0.00
	2	0.09	0.09	0.03	0.02	0.01	0.00
	3	0.24	0.18	0.06	0.07	0.07	0.02
	4	0.40	0.18	0.06	0.11	0.10	0.03
	5	0.38	0.18	0.06	0.21	0.14	0.04

**Two-sided t-tests**									
**Figure**	**Parameter**	**N**	**Mean**	**SEM**	**SD**	**Cohen's *d***	**t**	**df**	**P-Value**
Fig. 7b	SS Hz - WT - Anterior	23 cells, 3 mice	104.40	5.38	25.80				
	SS Hz - L7-KO - Anterior	25 cells, 3 mice	101.20	3.94	19.70	0.14	0.49	46	0.6270
Fig. 7c	SS CV - WT - Anterior	23 cells, 3 mice	0.46	0.02	0.08				
	SS CV - L7-KO - Anterior	25 cells, 3 mice	0.48	0.01	0.05	-0.32	1.19	46	0.2388
Fig. 7d	SS CV2 - WT - Anterior	23 cells, 3 mice	0.43	0.01	0.04				
	SS CV2 - L7-KO - Anterior	25 cells, 3 mice	0.47	0.01	0.04	-1.00	2.72	46	0.0092
Fig. 7f	SS Hz - WT - Posterior	25 cells, 3 mice	70.90	3.90	19.50				
	SS Hz - L7-KO - Posterior	21 cells, 3 mice	76.30	5.11	23.40	-0.25	0.85	45	0.3982
Fig. 7g	SS CV - WT - Posterior	25 cells, 3 mice	0.35	0.01	0.07				
	SS CV - L7-KO - Posterior	21 cells, 3 mice	0.49	0.02	0.09	-1.72	6.00	45	<0.0001
Fig. 7h	SS CV2 - WT - Posterior	25 cells, 3 mice	0.36	0.01	0.06				
	SS CV2 - L7-KO - Posterior	21 cells, 3 mice	0.47	0.01	0.06	-1.79	6.30	45	<0.0001
Fig. 7j	SS Hz - WT - Flocculus	23 cells, 3 mice	68.20	3.13	15.00				
	SS Hz - L7-KO - Flocculus	19 cells, 3 mice	69.60	4.31	18.78	-0.08	0.26	40	0.7986
Fig. 7k	SS CV - WT - Flocculus	23 cells, 3 mice	0.39	0.02	0.07				
	SS CV - L7-KO - Flocculus	19 cells, 3 mice	0.53	0.02	0.08	-1.87	5.51	40	<0.0001
Fig. 7l	SS CV2 - WT - Flocculus	23 cells, 3 mice	0.39	0.01	0.07				
	SS CV2 - L7-KO - Flocculus	19 cells, 3 mice	0.52	0.02	0.08	-1.73	5.75	40	<0.0001

**Mann-Whitney U-tests**									
**Figure**	**Parameter**	**N**	**Mean**	**SEM**	**SD**	**Cohen's *d***	**U**	**Sum of ranks**	**P-Value**
Fig. 8a	Time in chamber %, S1 (WT)	16 mice	53.72	4.96	19.86			358	
	Time in chamber %, empty (WT)	16 mice	19.86	4.35	17.41	1.82	34	170	0.0002
Fig. 8b	Time in chamber %, S1 (L7-KO)	17 mice	39.69	3.88	16.01			311	
	Time in chamber %, empty (L7-KO)	17 mice	37.32	3.61	14.90	0.15	131	284	0.6520
Fig. 8d	Time in chamber %, S1 (WT)	16 mice	26.38	2.14	8.56			142	
	Time in chamber %, S2 (WT)	16 mice	52.18	2.38	9.53	-2.85	6	386	0.0001
Fig. 8e	Time in chamber %, S1 (L7-KO)	17 mice	37.71	2.90	11.95			249	
	Time in chamber %, S2 (L7-KO)	17 mice	44.51	2.97	12.26	-0.56	96	346	0.0987
Fig. 8i	n of repeats (WT)	16 mice	2.56	0.33	1.32			191.5	
	n of repeats (L7-KO)	17 mice	4.00	0.24	1.00	-1.24	55.5	369	0.0023

**Two-sided t-tests**									
**Figure**	**Parameter**	**N**	**Mean**	**SEM**	**SD**	**Cohen's *d***	**t**	**df**	**P-Value**
Fig. 8c	Preference index S1-empty (WT)	16 mice	29.44	8.90	35.61				
	Preference index S1-empty (L7-KO)	17 mice	2.37	6.85	28.25	0.85	2.426	31	0.0213
Fig. 8f	Preference index S2-S1 (WT)	16 mice	25.80	4.29	17.14				
	Preference index S2-S1 (L7-KO)	17 mice	6.81	5.77	23.78	0.93	2.61	31	0.0136
Fig. 8g	Marbles Buried % (WT)	16 mice	55.47	5.52	22.06				
	Marbles Buried % (L7-KO)	17 mice	55.59	4.70	19.40	-0.01	0.02	31	0.9869
Fig. 8h	Time grooming seconds (WT)	13 mice	286.70	21.94	79.12				
	Time grooming seconds (L7-KO)	16 mice	232.80	16.25	65.01	0.75	2.02	27	0.0540

Two-sided t-tests

Figure	Parameter	N	Mean	SEM	SD	Cohen's *d*	t	df	P-Value
Supp. Fig. 2a	Na spike amp. mV (WT)	7 cells, 6 mice	51.78	2.40	6.35				
	Na spike amp. mV (KO)	9 cells, 4 mice	48.45	1.98	5.94	0.54	1.08	14	0.2989
Supp. Fig. 2c	First Ca spike amp. (WT)	7 cells, 6 mice	31.81	4.52	11.95				
	First Ca spike amp. (KO)	9 cells, 4 mice	33.92	2.12	6.35	-0.23	0.46	14	0.6546

Mann-Whitney U-tests

Figure	Parameter	N	Mean	SEM	SD	Cohen's *d*	U	Sum of ranks	P-Value
Supp. Fig. 2b	No. of Spikelets (first CS) (WT)	7 cells, 6 mice	1.57	0.20	0.53			49	
	No. of Spikelets (first CS) (KO)	9 cells, 4 mice	2.00	0.24	0.71	-0.69	21	87	0.2349
Supp. Fig. 2d	No. of climbing fibre responses P9-P10 (WT)	10 cells, 3 mice	2.00	0.15	0.47			101.5	
	No. of climbing fibre responses P9-P10 (KO)	12 cells, 3 mice	2.25	0.13	0.45	-0.54	46.5	151	0.2431
Supp. Fig. 2e	No. of climbing fibre responses P25-P35 (WT)	5 cells, 3 mice	1.00	0.00	0.00				
	No. of climbing fibre responses P25-P35 (KO)	12 cells, 3 mice	1.00	0.00	0.00	-	-	-	1
Supp. Fig. 2f	CS paired pulse depression P9-P10 (WT)	9 cells, 3 mice	0.59	0.05	0.14			89	
	CS paired pulse depression P9-P10 (KO)	8 cells, 3 mice	0.54	0.04	0.11	0.39	28	64	0.4807
Supp. Fig. 2g	CS paired pulse depression P25-P35 (WT)	5 cells, 3 mice	0.75	0.05	0.11			37	
	CS paired pulse depression P25-P35 (KO)	11 cells, 3 mice	0.77	0.03	0.10	-0.21	22	99	0.5711

Two-sided t-tests									
**Figure**	**Parameter**	**N**	**Mean**	**SEM**	**SD**	**Cohen's *d***	**t**	**df**	**P-Value**
Supp. Fig. 3a	Complex Spikes Hz (WT) - Anterior	23 cells, 3 mice	1.28	0.06	0.29				
	CS Hz (KO) - Anterior	26 cells, 3 mice	1.32	0.05	0.24	-0.12	0.42	47	0.6780
Supp. Fig. 3b	CS pause (WT) - Anterior	23 cells, 3 mice	9.11	0.41	1.98				
	CS pause (KO) - Anterior	26 cells, 3 mice	10.00	0.56	2.87	-0.37	1.24	47	0.2210
Supp. Fig. 3c	CS Hz (WT) - Posterior	16 cells, 3 mice	0.67	0.05	0.19				
	CS Hz (KO) - Posterior	27 cells, 3 mice	0.84	0.07	0.37	-0.61	1.71	41	0.0942
Supp. Fig. 3d	CS pause (WT) - Posterior	16 cells, 3 mice	19.66	1.38	5.52				
	CS pause (KO) - Posterior	27 cells, 3 mice	17.61	1.02	5.29	0.38	1.21	41	0.2338

Two-sided t-tests

Figure	Parameter	N	Mean	SEM	SD	Cohen's *d*	t	df	P-Value
Supp. Fig. 5a	Speed cm/s (WT)	15 mice	12.28	0.72	2.79				
	Speed cm/s (L7-KO)	17 mice	13.24	0.63	2.61	-0.36	1.00	30	0.3240
Supp. Fig. 5b	Distance m (WT)	15 mice	7.37	0.43	1.68				
	Distance m (L7-KO)	17 mice	7.94	0.38	1.57	-0.35	1.00	30	0.3240

Erasmus Ladder - Descriptive Statistics

		Day 1			Day 2		
**Figure**	**Genotype**	**Mean**	**SEM**	**SD**	**Mean**	**SEM**	**SD**
Supp. Fig. 5e	WT	0.329	0.083	0.203	0.471	0.063	0.155
	L7-KO	0.242	0.062	0.186	0.331	0.071	0.213
Supp. Fig. 5f	WT	410.62	42.96	105.23	330.75	20.15	49.37
	L7-KO	404.42	32.86	98.58	359.26	28.06	84.17

OKR - Descriptive Statistics

Figure	Genotype	Hz	Mean	SEM	SD
Supp. Fig. 5g	WT	0.1	0.90	0.01	0.11
		0.2	0.89	0.01	0.07
		0.4	0.69	0.01	0.10
		0.6	0.48	0.01	0.08
		0.8	0.34	0.01	0.09
		1	0.32	0.01	0.05
	L7-KO	0.1	0.97	0.01	0.12
		0.2	0.90	0.01	0.07
		0.4	0.70	0.01	0.09
		0.6	0.50	0.01	0.09
		0.8	0.34	0.01	0.07
		1	0.28	0.00	0.04
Supp. Fig. 5h	WT	0.1	-0.18	0.22	1.97
		0.2	-5.42	0.25	2.29
		0.4	-8.30	0.13	1.16
		0.6	-17.10	0.49	4.42
		0.8	-23.39	0.21	1.90
		1	-26.58	0.41	3.72
	L7-KO	0.1	1.22	0.45	4.01
		0.2	-5.02	0.12	1.08
		0.4	-8.19	0.18	1.61
		0.6	-15.43	0.29	2.64
		0.8	-24.63	0.59	5.31
		1	-29.51	0.79	7.12

VOR - Descriptive Statistics

Figure	Genotype	Hz	Mean	SEM	SD
Supp. Fig. 5i	WT	0.1	0.29	0.01	0.09
		0.2	0.35	0.02	0.15
		0.4	0.47	0.01	0.11
		0.6	0.68	0.02	0.14
		0.8	0.81	0.02	0.13
		1	0.89	0.01	0.11
	L7-KO	0.1	0.35	0.01	0.12
		0.2	0.38	0.01	0.10
		0.4	0.53	0.01	0.13
		0.6	0.72	0.01	0.05
		0.8	0.81	0.01	0.10
		1	0.89	0.01	0.07
Supp. Fig. 5j	WT	0.1	52.42	2.45	19.57
		0.2	30.88	2.03	16.27
		0.4	27.87	1.21	9.65
		0.6	19.81	0.50	4.03
		0.8	12.47	0.43	3.47
		1	10.01	0.29	2.36
	L7-KO	0.1	42.67	1.49	13.45
		0.2	22.37	1.08	9.68
		0.4	24.22	0.70	6.28
		0.6	17.95	0.51	4.62
		0.8	13.31	0.47	4.24
		1	9.80	0.38	3.38

Repeated Measures ANOVAs

Figure	Outcome Variable	N	Parameter	Type III sum of squares	df	mean square	F	P-Value	Partial η2
Supp. Fig. 5c	Speed (cm/s)	WT: 16 mice; L7-KO: 16							
			Intercept	10.704.7	1	10.704.7	282.41		
			Genotype	58.0	2	29.0	0.77	0.4850	0.105
			Error	492.8	13	37.9			
Supp. Fig. 5d	Distance (cm)	WT: 16 mice; L7-KO: 16							
			Intercept	36.558.175.2	1	36.558.175.2	297.89		
			Genotype	184.094.4	2	92.047.2	0.75	0.4920	0.103
			Error	1.595.384.6	13	122.721.9			
Supp. Fig. 5e	Efficient steps (%)	WT: 10 mice; L7-KO: 6							
			Intercept	3.4	1	3.4	47.68		
			Genotype	0.1	1	0.1	1.31	0.2740	0.091
			Error	0.9	13	0.1			
Supp. Fig. 5f	Timing steps (ms)	WT: 10 mice; L7-KO: 6							
			Intercept	4.076.701.9	1	4.076.701.9	426.58		
			Genotype	903.4	1	903.4	0.10	0.7630	0.007
			Error	124.236.3	13	9.556.6			
Supp. Fig. 5g	OKR Gain	WT: 9 mice; L7-KO: 9							
			Intercept	40.2	1	40.2	1667.91		
			Genotype	0.0	1	0.0	0.24	0.6290	0.015
			Error	0.4	16	0.0			
Supp. Fig. 5h	OKR Phase	WT: 9 mice; L7-KO: 9							
			Intercept	19.814.7	1	19.814.7	857.20		
			Genotype	0.3	1	0.3	0.01	0.9180	0.001
			Error	369.9	16	23.1			
Supp. Fig. 5i	VOR Gain	WT: 8 mice; L7-KO: 9							
			Intercept	36.3	1	36.3	888.21		
			Genotype	0.0	1	0.0	0.67	0.4260	0.043
			Error	0.6	15	0.0			
Supp. Fig. 5j	VOR Phase	WT: 8 mice; L7-KO: 9							
			Intercept	56.846.4	1	56.846.4	290.05		
			Genotype	378.6	1	378.6	1.93	0.1850	0.114
			Error	2.939.8	15	196.0			

Two-sided t-tests									
**Figure**	**Parameter**	**N**	**Mean**	**SEM**	**SD**	**Cohen's *d***	**t**	**df**	**P-Value**
Supp. Fig. 6a	Complex Spikes Hz (WT) - Anterior	23 cells, 3 mice	0.92	0.10	0.48				
	CS Hz (L7-KO) - Anterior	25 cells, 3 mice	0.85	0.06	0.30	0.18	0.59	46	0.5562
Supp. Fig. 6b	CS pause (WT) - Anterior	23 cells, 3 mice	15.40	1.08	5.20				
	CS pause (L7-KO) - Anterior	25 cells, 3 mice	14.10	0.72	3.60	0.30	0.98	46	0.3302
Supp. Fig. 6c	CS Hz (WT) - Posterior	25 cells, 3 mice	0.87	0.05	0.24				
	CS Hz (L7-KO) - Posterior	21 cells, 3 mice	0.96	0.09	0.40	-0.28	0.97	44	0.3391
Supp. Fig. 6d	CS pause (WT) - Posterior	25 cells, 3 mice	22.90	1.42	7.10				
	CS pause (L7-KO) - Posterior	21 cells, 3 mice	20.20	2.01	9.20	0.33	1.12	44	0.2673
Supp. Fig. 6e	CS Hz (WT) - flocculus	23 cells, 2 mice	1.12	0.08	0.40				
	CS Hz (L7-KO) - flocculus	19 cells, 2 mice	1.25	0.08	0.37	-0.34	1.11	40	0.2753
Supp. Fig. 6f	CS pause (WT) - flocculus	23 cells, 2 mice	23.20	1.49	7.13				
	CS pause (L7-KO) - flocculus	19 cells, 2 mice	24.76	1.64	7.14	-0.22	0.70	40	0.4908

Chi-square test						
**Figure**	**Parameter**	**N**	**%**	**Chi-square**	**df**	**P-Value**
Supp. Fig. 7a	Time spent (% inner/middle/outer zone) - WT	17 mice	76/20/4			
	Time spent (% inner/middle/outer zone) - L7-KO	15 mice	71/24/5	0.645	2	0.7244

Two-sided t-tests									
**Figure**	**Parameter**	**N**	**Mean**	**SEM**	**SD**	**Cohen's *d***	**t**	**df**	**P-Value**
Supp. Fig. 7b	Latency to food (s) - WT	5 mice	378.80	133.20	297.90				
	Latencyto food (s) - L7-KO	10 mice	289.60	102.50	324.20	0.29	0.51	13	0.6153

Germline deletion test

Univariate GLM: Three chamber social interaction task (Fig. 8c)										
**Outcome Variable**	**N**	**Parameter**	**Type III sum of squares**	**df**	**mean square**	**F**	**P-Value**	**R squared**	**Adjusted R squared**
Preference index (S1 vs Empty)	WT: 16, L7-KO: 17	Corrected Model	8.434	3	2.811	2.77	0.059	0.223	0.143
		Intercept	8.878	1	8.878	8.76	0.006		
		non-specific deletion	2.396	1	2.396	2.36	0.135		
		genotype	7.932	1	7.932	7.83	0.009		
		non-specific deletion * genotype	54	1	54	0.05	**0.818°**		
		Error	29.396	29	1.014				
		Total	45.752	33					
		Corrected Total	37.830	32					
		**°Interaction not significant: remove**							
		Corrected Model	8.379	2	4.190	4.27	0.023	0.222	0.170
		Intercept	9.656	1	9.656	9.84	0.004		
		non-specific deletion	2.342	1	2.342	2.39	**0.133°**		
		genotype	8.022	1	8.022	8.17	0.008		
		Error	29.451	30	982				
		Total	45.752	33					
		Corrected Total	37.830	32					
		**°Non-specific deletion not significant: remove**							
		Corrected Model	6.037	1	6.037	5.89	0.021	0.160	0.132
		Intercept	8.339	1	8.339	8.13	0.008		
		genotype	6.037	1	6.037	5.89	**0.021°**		
		Error	31.793	31	1.026				
		Total	45.752	33					
		Corrected Total	37.830	32					
		**°Genotype significant**							

Univariate GLM: Three chamber social interaction task (Fig. 8f)										
**Outcome Variable**	**N**	**Parameter**	**Type III sum of squares**	**df**	**mean square**	**F**	**P-Value**	**R squared**	**Adjusted R squared**
Preference index (S1 vs S2)	WT: 16, L7-KO: 17	Corrected Model	3.063	3	1.021	2.22	0.108	0.186	0.102
		Intercept	7.963	1	7.963	17.28	0		
		non-specific deletion	69	1	69	0.15	0.702		
		genotype	2.893	1	2.893	6.28	0.018		
		non-specific deletion * genotype	34	1	34	0.07	**0.788°**		
		Error	13.364	29	461				
		Total	24.892	33					
		Corrected Total	16.426	32					
		**°Interaction not significant: remove**							
		Corrected Model	3.029	2	1.514	3.39	0.047	0.184	0.130
		Intercept	8.745	1	8.745	19.58	0		
		non-specific deletion	56	1	56	0.13	**0.726°**		
		genotype	2.893	1	2.893	6.48	0.016		
		Error	13.398	30	447				
		Total	24.892	33					
		Corrected Total	16.426	32					
		**°Non-specific deletion not significant: remove**							
		Corrected Model	2.973	1	2.973	6.85	0.014	0.181	0.155
		Intercept	8.764	1	8.764	20.20	0		
		genotype	2.973	1	2.973	6.85	**0.014°**		
		Error	13.454	31	434				
		Total	24.892	33					
		Corrected Total	16.426	32					
		**°Genotype significant**							

Germline deletion test

Univariate GLM: T-Maze (Fig. 8i)									
**Outcome Variable**	**N**	**Parameter**	**Type III sum of squares**	**df**	**mean square**	**F**	**P-Value**	**R squared**	**Adjusted R squared**
Repetition	WT: 16, L7-KO: 17	Corrected Model	19.0	3	6.34	4.60	0.009	0.323	0.253
		Intercept	308.4	1	308.43	223.92	0		
		non-specific deletion	2.0	1	1.98	1.44	0.24		
		genotype	10.8	1	10.83	7.86	0.009		
		non-specific deletion * genotype	0.0	1	0.02	0.01	**0.913°**		
		Error	39.9	29	1.38				
		Total	419.0	33					
		Corrected Total	59.0	32					
		**°Interaction not significant: remove**							
		Corrected Model	19.0	2	9.50	7.14	0.003	0.322	0.277
		Intercept	353.5	1	353.54	265.41	0		
		non-specific deletion	2.0	1	1.98	1.48	**0.233°**		
		genotype	11.5	1	11.54	8.67	0.006		
		Error	40.0	30	1.33				
		Total	419.0	33					
		Corrected Total	59.0	32					
		**°Non-specific deletion not significant: remove**							
		Corrected Model	17.0	1	17.03	12.59	0.001	0.289	0.266
		Intercept	355.0	1	354.97	262.39	0		
		genotype	17.0	1	17.03	12.59	**0.001°**		
		Error	41.9	31	1.35				
		Total	419.0	33					
		Corrected Total	59.0	32					
		**°Genotype significant**							
